# Inhibition of BRD4 prevents proliferation and epithelial–mesenchymal transition in renal cell carcinoma via NLRP3 inflammasome-induced pyroptosis

**DOI:** 10.1038/s41419-020-2431-2

**Published:** 2020-04-17

**Authors:** Yi-Fan Tan, Min Wang, Zhi-Yuan Chen, Lei Wang, Xiu-Heng Liu

**Affiliations:** Department of Urology, Renmin Hospital of Wuhan University, 430060 Wuhan, Hubei China

**Keywords:** Cancer therapy, Renal cell carcinoma, Cell death

## Abstract

BRD4 has long been implicated in many different pathological processes, in particular, the development of cancer and inflammation. Pyroptosis is a newly recognized type of inflammatory programmed cell death. However, the correlation between BRD4 and pyroptosis in renal cell carcinoma (RCC) remains elusive. The present study demonstrates that BRD4 expression levels are markedly upregulated, while pyroptosis-associated proteins are significantly reduced, in RCC tissues and cells. Inhibition of BRD4, via either genetic knockdown or use of bromodomain inhibitor JQ1, prevented cell proliferation and epithelial–mesenchymal transition (EMT) progression and induced caspase-1-dependent pyroptosis in RCC both in vitro and in vivo. In addition, BRD4 inhibition suppressed proliferation and EMT though pyroptosis in vitro and in vivo. Moreover, NLRP3, which mediates caspase-1-dependent pyroptosis, was increased upon BRD4 inhibition. Furthermore, the transcriptional activity of NLRP3 was enhanced by BRD4 inhibition, and this enhancement was blocked by activation of NF-κB phosphorylation, indicating that NF-κB is an upstream regulator of NLRP3. Collectively, these results show that BRD4 inhibition prevents cell proliferation and EMT, and exerts an antitumor effect in RCC by activating the NF-κB–NLRP3–caspase-1 pyroptosis signaling pathway. Thus, BRD4 is a potential target for RCC treatment, and JQ1 shows promise as a therapeutic agent for this disease.

## Introduction

Kidney cancer is an important public health concern, with an estimated 0.338 million new cases and 14,4000 deaths per year worldwide^1^. RCC, which accounts for ~85% of such malignancies, is the sixth most common cancer in males and eighth most common in females in the United States^2,3^. Established risk factors for this malignancy include obesity, overweight, smoking, and mutations in specific genes^4,5^. Evidence indicates that surgery is the only curative treatment for localized RCC. Unfortunately, approximately one-third of patients treated with surgery experience relapse in distant sites, and the overall prognosis is poor once the disease progresses^6,7^. Thus, a detailed understanding of tumor biology will help provide novel therapeutic strategies for patients with RCC.

The bromodomain and extra terminal domain (BET) family of proteins consists of epigenetic readers, including BRD2, BRD3, BRD4, and BRDT. Through their N-terminal bromodomains, BET family proteins bind to acetylated lysine residues of histone tails, change chromatin structure, and exert an important influence on diverse physiological processes^8^. Abnormal expression of BET proteins has been reported to be involved in many different pathological processes, especially in the development of cancer and inflammation^9,10^. Therefore, inhibition of BET proteins may be a promising therapeutic strategy for many diseases. BET inhibitor JQ1, a relative specificity inhibitor of BRD4, binds to the bromodomain pocket in competitively with acetylated peptide binding, leading to substitution of BET proteins and transcriptional regulatory complexes from acetylated chromatin^11,12^. Recent studies have shown that JQ1 has a significant role in cancer and inflammatory response^13–15^. Our previous study demonstrated that BRD4 inhibition suppressed tumor growth in prostate cancer via the enhancement of FOXO1^16^. A recent study indicated that inhibition of BRD4 by JQ1 could suppress vascular inflammation though inhibiting NF-κB activation^17^. Another study reported that BRD4 inhibition attenuates pro-inflammatory cytokines produced in the microglia, thereby promoting functional recovery after spinal cord injury^18^. Deficiency of BRD4 has been reported to induce apoptosis and inhibit cell proliferation in RCC cells^19^. However, the association between BRD4 and tumor-related inflammation in RCC remains unknown and the underlying molecular mechanisms have not been studied.

Pyroptosis, a newly recognized type of programmed inflammatory cell death, can be activated by canonical caspase-1 inflammasomes or non-canonical caspase-4-, caspase-5-, and caspase-11-mediated pathways^20^. When pyroptosis occurs via canonical signaling, caspase-1 is converted into its active forms (p20 and p10 subunits) by inflammasomes (NLRP3, AIM2, etc.) and then activates pro-inflammatory cytokines interleukin (IL)-18 and IL-1β to mature IL-18 and IL-1β; these have strong pro-inflammatory activity and promote vasodilation and extravasation of cells. Finally, the cells swell, burst, and eventually die^21–23^. In the non-canonical pathway, lipopolysaccharide binds directly to caspases 4, 5, and 11 to induce pyroptosis^24^. Previous studies have demonstrated that pyroptosis aggravates hepatic fibrosis diabetes and diabetic cardiomyopathy^25,26^. A recent study of tumor cells showed that induction of caspase-1-mediated pyroptosis by simvastatin in non-small-cell lung cancer (NSCLC) promoted cell death and exerted antitumor effects^27^. Caspase-1 is downregulated in many cancer types, and its loss enhances tumor formation and promotes cancer development^27,28^. However, the role of caspase-1-dependent pyroptosis in RCC remains unknown.

In the present study, we determined that BRD4 was upregulated whereas caspase-1 were downregulated in RCC cancer samples and cell lines. Inhibition of BRD4 suppressed proliferation and metastasis, and promote pyroptosis in RCC. Our data indicated that the potential mechanisms of BRD4 inhibition might involve blocking NF-κB signaling and activating NLRP3 inflammasome-elicited pyroptosis.

## Results

### Overexpression of BRD4 and downregulation of caspase-1 and IL-1β in RCC tissue specimens and cell lines

**Fig. 1:**
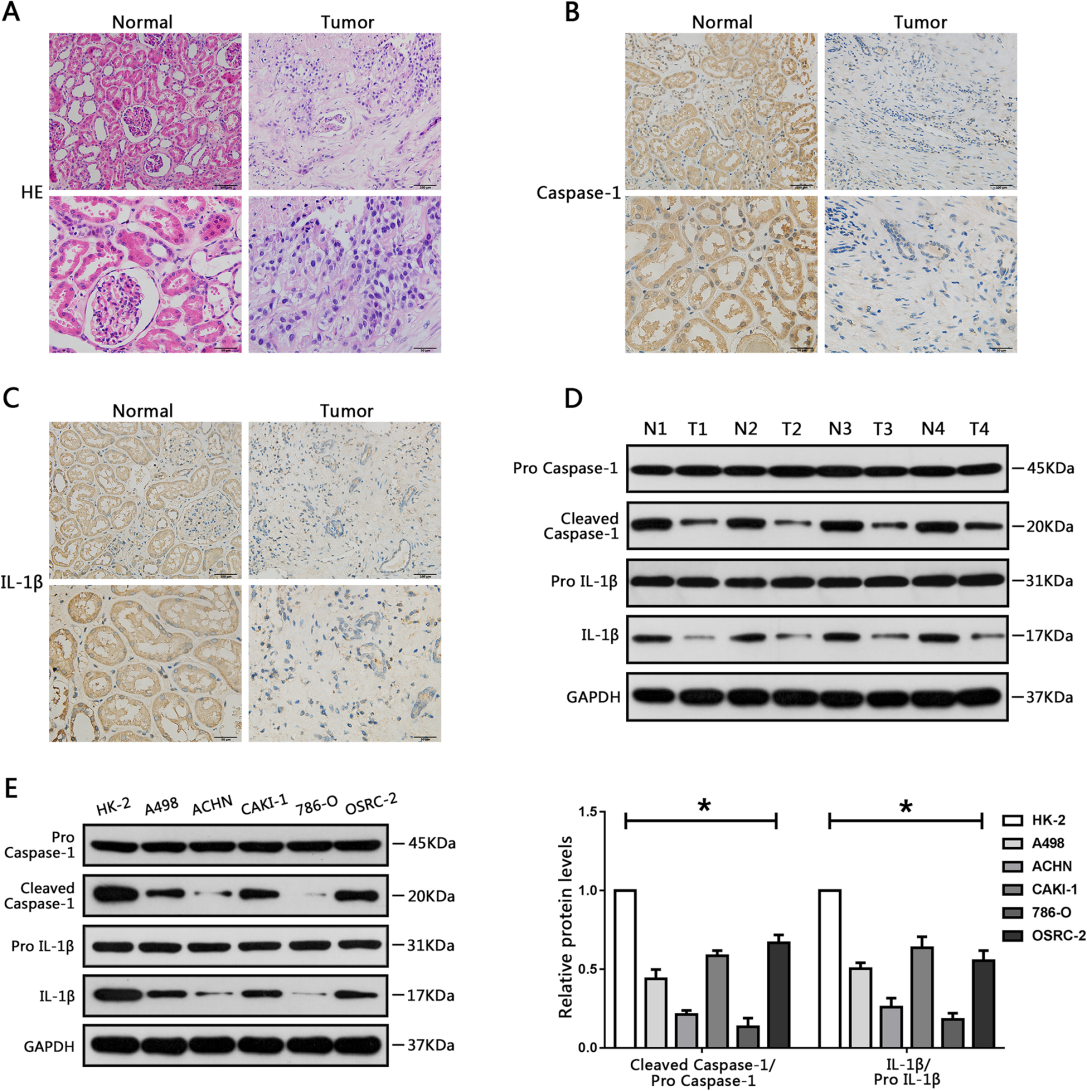
Downregulation of Caspase-1 and IL-1β in RCC tissue specimens and cell lines. **a** Hematoxylin and eosin staining of renal cell carcinoma (RCC) specimens and adjacent normal renal tissues. **b** Immunohistochemical staining of caspase-1 in RCC specimens and adjacent normal renal tissues. **c** Immunohistochemical analysis of IL-1β in RCC tissues and adjacent normal renal tissues. **d** Western blot analysis of expression levels of caspase-1 and IL-1β in RCC tissues and surrounding normal regions. **e** Relative caspase-1 and IL-1β protein levels in HK-2 cells (human renal tubule epithelial cell line), and five RCC cell lines (A498, ACHN, CAKI-1, 786-O, and OSRC-2) were analyzed using western blot analysis. Results are shown as means ± SD. *P < 0.05, relative to the HK-2 cell line.

We first used HE staining to detect renal tissue morphology in RCC and surrounding non-tumor tissues (Fig. 1a). Then RCC tissue samples and adjacent non-tumor samples were collected and assessed by immunohistochemistry. The results showed that BRD4 was stained more intensely in tumor samples than in non-tumor samples (Fig. S1A). We further determined BRD4 expression in RCC cells (A498, 786-O, ACHN, CAKI-1, and OSRC-2) and human normal renal tubule epithelial cell line (HK-2). Real-time PCR (RT-PCR) results showed that the expression of BRD4 was increased in RCC cells compared with HK-2 cells (Fig. S1B).

Pyroptosis-associated proteins such as caspase-1 and IL-1β have been reported to be downregulated in several cancer types^27,28^. Consistent with this, in our immunohistochemical staining experiments, caspase-1 and IL-1β in RCC cancer tissues were stained more lightly than in non-tumor tissues (Fig. 1b, c). Further, western blot assays showed that caspase-1 and IL-1β expression levels were downregulated in RCC cancer tissues, indicating the same trend (Fig. 1d). Western blot assays were also performed to detect the difference in caspase-1 and IL-1β protein expression between RCC cells (A498, ACHN, CAKI-1, 786-O, and OSRC-2) and HK-2 cells. As expected, caspase-1 and IL-1β expression were also downregulated in RCC cells relative to HK-2 cells (Fig. 1e). Taken together, these results indicate that BRD4 and pyroptosis might be involved in the initiation of RCC.

### BRD4 inhibition suppresses cellular proliferation and EMT progression in RCC cell lines

**Fig. 2:**
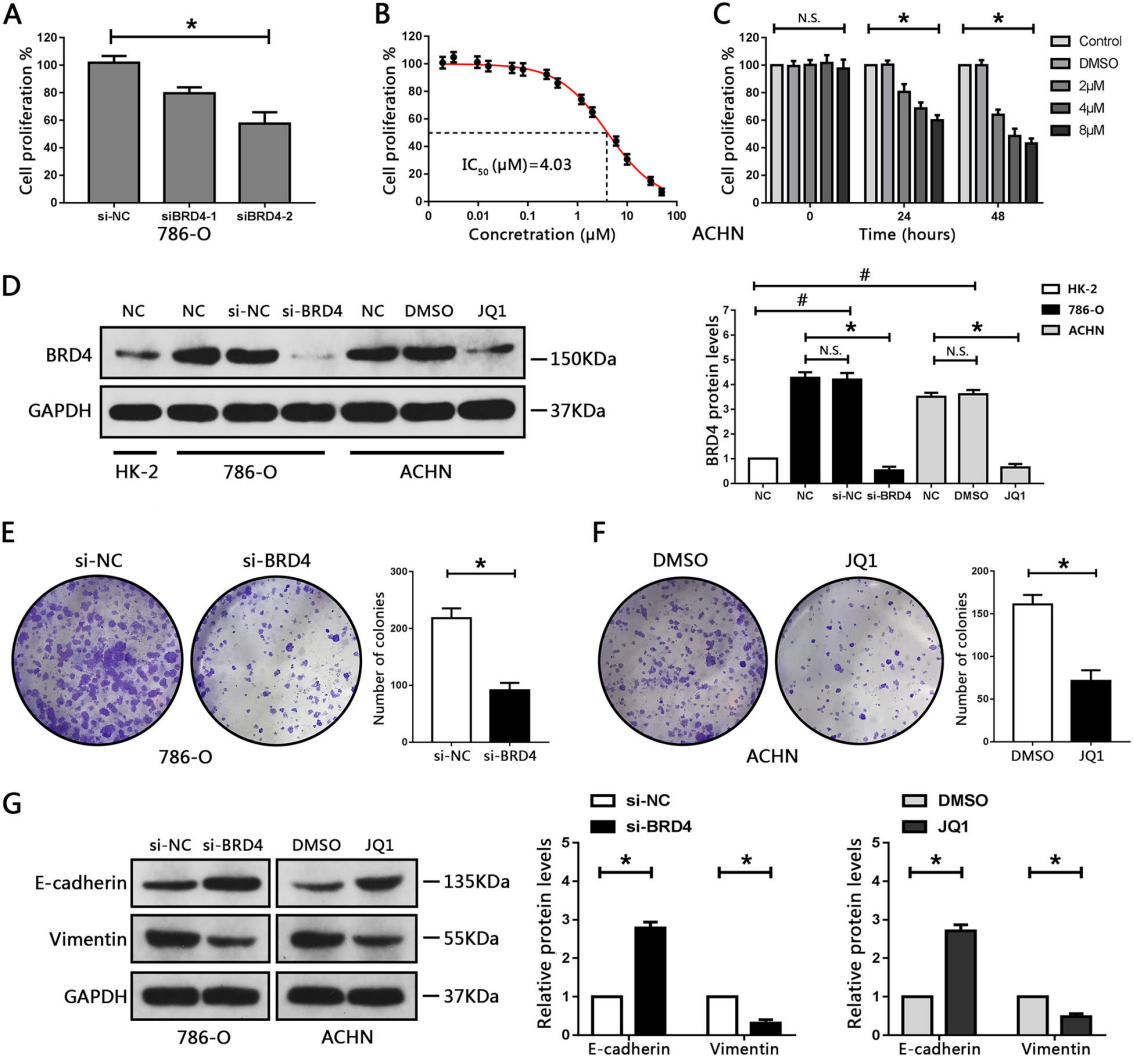
BRD4 inhibition suppressed cellular proliferation and EMT progression in RCC cell lines. **a** Cell proliferation of 786-O cells transfected with BRD4-targeted siRNA was evaluated using CCK8 assay. *P < 0.05, relative to the si-NC group. **b** Cell proliferation of ACHN cells treated with JQ1 (1.92, 3.2, 9.6, 16, 48, 80, 240, 400, 1.2, 2, 6, 10, 30, and 50 μM) for 48 h was determined using the CCK8 assay, and the corresponding half-maximal inhibitory concentration (IC_50_) values were determined. **c** Cell proliferation of ACHN cells after treatment with various concentrations of JQ1 for 0, 24, and 48 h was evaluated using the CCK8 assay. *P < 0.05 vs. DMSO. **d** Western blot analysis of BRD4 levels following transfection with si-BRD4 or treatment with JQ1 for 48 h. *P < 0.05, relative to the si-NC group or the DMSO group. ^#^*P* < 0.05, relative to HK-2 cells. N.S. *P* > 0.05 vs. control. **e**, **f** Colony formation ability of 786-O and ACHN cells transfected with siBRD4 or treated with JQ1, and representative images. *P < 0.05 vs. si-NC or DMSO. **g** Protein levels of E-cadherin and vimentin in 786-O and ACHN cells transfected with siBRD4 or treated with JQ1, respectively, were assessed by western blotting. Experiments were performed in triplicate. *P < 0.05 vs. si-NC or DMSO.

The 786-O cells exhibited much higher expression levels of BRD4 than the other four RCC cell lines (Fig. S1B). Thus, the 786-O cell line was employed for BRD4 knockdown analysis to determine the biological effects of BRD4 in RCC cells. BRD4 expression levels were verified by quantitative RT-PCR and western blot assays in 786-O cell lines, showing that BRD4 expression levels were significantly decreased after transfection with BRD4-targeted short interfering RNA (siRNA) (Fig. S2A, B). Furthermore, we observed that knockout of BRD4 expression notably attenuated cell proliferation in 786-O cells compared with negative controls (Fig. 2a).

To avoid the toxicity of JQ1 to normal renal cells, HK-2 cells were treated with different concentrations of JQ1. CCK8 assays were used to measure cell proliferation, the results showed that treatment with JQ1 did not attenuate cell proliferation in HK-2 cells, suggesting that JQ1 has no obvious toxicity to renal tubule epithelial cells (Fig. S2C, D).

Next, we treated RCC cells with various concentrations of JQ1, showing that JQ1 treatment decreased cell proliferation in a dose- and time-dependent manner; IC_50_ values were calculated for each cell line (Figs. 2b, c and S2E–L). ACHN cells were more sensitive than the other four RCC cell lines, with an IC_50_ value of 4.03 µM. Therefore, ACHN cells were selected for all subsequent JQ1 treatment assays. ACHN cells treated with JQ1 showed a marked decrease in BRD4 levels (Fig. 2d). In addition, colony formation assays showed that inhibition of BRD4, either by siBRD4 or JQ1, impaired the colony formation capacity of RCC cells (Fig. 2e, f). BRD4 has also been reported to promote metastasis in cancer cells^29^. Next, we evaluated whether BRD4 could influence EMT progression in RCC; inhibition of BRD4 obviously suppressed the migration and invasion of RCC cells (Fig. S3A–D). Furthermore, the expression of epithelial-related protein, E-cadherin, was significantly increased, whereas mesenchymal-associated protein, vimentin was obviously downregulated after BRD4 silencing or JQ1 treatment (Fig. 2g). Collectively, we considered that inhibition of BRD4 suppresses cell proliferation and EMT progression in RCC cells.

### BRD4 inhibition activates caspase-1-dependent pyroptosis in vitro

**Fig. 3:**
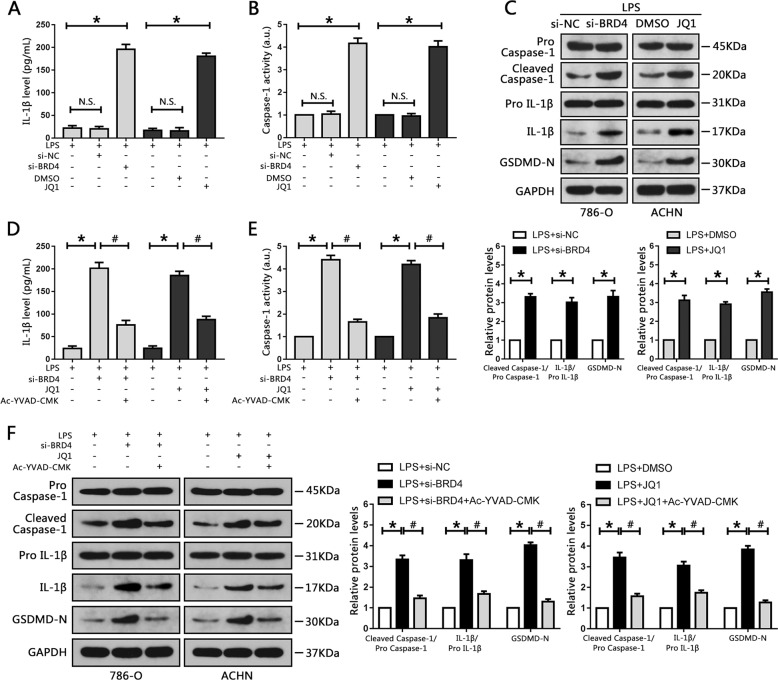
BRD4 inhibition activated caspase-1-dependent pyroptosis in RCC cell lines. **a** 786-O and ACHN cells were pretreated with LPS though the methods mention above, then transfected with siBRD4 or treated with JQ1, respectively, and the levels of IL-1β secreted into the culture medium were assessed using ELISA. **b** Activity of caspase-1 following siBRD4 transfection and JQ1 treatment was measured. **c** Western blots of caspase-1, IL-1β and GSDMD levels in 786-O and ACHN cells following transfection with siBRD4 or treatment with JQ1, respectively. The bar graph shows the relative levels of caspase-1, IL-1β and GSDMD from three independent experiments. **d–f** 786-O and ACHN cells were treated with 50 μM Ac-YVAD-CMK for 24 h, then transfected with siBRD4 or treated with JQ1. **d** Production of IL-1β secreted into the culture medium was measured using ELISA. **e** Caspase-1 activity was evaluated in 786-O and ACHN cells. **f** Western blot analysis of caspase-1, IL-1β and GSDMD levels. The bar graph shows the relative levels of caspase-1 and IL-1β from three independent experiments. *P < 0.05 vs. control; ^#^*P* < 0.05 vs. si-BRD4 or JQ1; N.S. *P* > 0.05 vs. control.

Previous studies have reported that BRD4 is involved in the development of cancer and inflammation^9^. BRD4 deficiency was also shown to induce apoptosis in RCC cell lines^19^, but whether BRD4 knockout could induce pyroptosis in RCC cells was still unclear. Here, 786-O cells and ACHN cells were pre-treated with LPS to activate pyroptosis, then transfected with BRD4 target-siRNA or treated with JQ1, respectively. An ELISA assay was carried out to detect the production of pyroptosis-associated markers (IL-1β) in the culture medium. Caspase-1 activity was also examined. The production of IL-1β in the culture medium was significantly increased after transfection with siBRD4 or JQ1 treatment (Fig. 3a). Caspase-1 activity was also increased relative to the control groups (Fig. 3b). Intracellular protein expression levels of IL-1β, caspase-1 and GSDMD were also evaluated; BRD4 inhibition significantly increased IL-1β, cleaved caspase-1 and cleaved N-terminal GSDMD levels compared with control groups in RCC cells (Fig. 3c). In addition, flow cytometry was used to determine cell pyroptosis and the results revealed that inhibition of BRD4 in RCC cells significantly increased the levels of cell pyroptosis (Fig. S4A).

To further investigate whether pyroptosis induced by BRD4 inhibition was mediated by caspase-1, Ac-YVAD-CMK, a specific caspase-1 inhibitor that suppresses caspase-1 activation and expression, was employed in this study. Suppression of caspase-1 by this pharmaceutical inhibitor attenuated BRD4 knockdown-induced IL-1β upregulation and decreased caspase-1 and GSDMD activation in 786-O cells (Fig. 3d–f). A combination of Ac-YVAD-CMK and JQ1 also resulted in downregulation of IL-1β, caspase-1 and GSDMD activation in ACHN cells (Fig. 3d–f). The results from flow cytometry indicated that cell pyroptosis levels were attenuated when treated with Ac-YVAD-CMK (Fig. S4B). Therefore, BRD4 inhibition can induce caspase-1-dependent pyroptosis in RCC cells.

### Pyroptosis activation mediates BRD4 inhibition-elicited suppression of cell proliferation and EMT in vitro

**Fig. 4:**
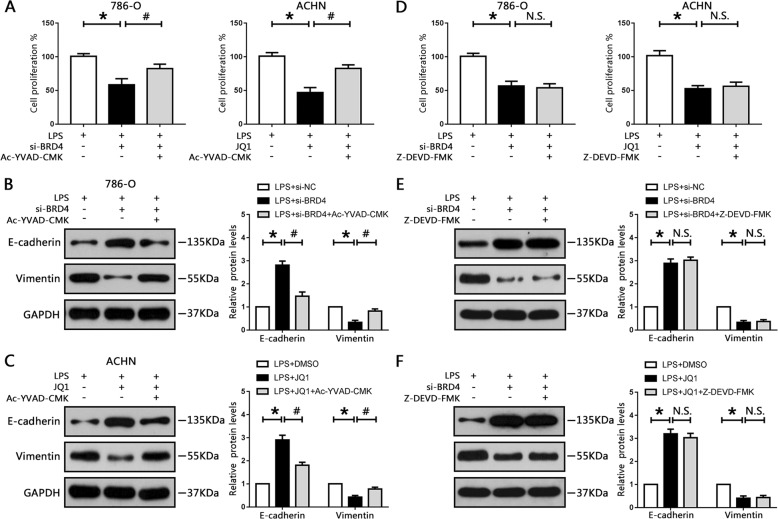
Pyroptosis activation mediated BRD4 inhibition-induced suppression of cell proliferation and EMT in RCC cells. 786-O cells were pre-treated with 50 μM Ac-YVAD-CMK or 50 μM Z-DEVD-FMK for 24 h, then transfected with siBRD4; ACHN cells were pre-treated with 50 μM Ac-YVAD-CMK or 50 μM Z-DEVD-FMK for 24 h, then treated with JQ1. **a**, **d** Cell proliferation of 786-O and ACHN cells was measured using CCK8 assay. **b**, **c**, **e**, **f** Protein levels of E-cadherin and vimentin were measured by western blotting and normalized to GAPDH. Experiments were performed in triplicate. *P < 0.05 vs. control; ^#^*P* < 0.05 vs. si-BRD4 or JQ1; N.S. *P* > 0.05 vs. si-BRD4 or JQ1.

Recent studies have indicated that pyroptosis is involved in cell proliferation and motility in NSCLC and hepatocellular carcinoma^27,28^. Thus, we explored whether BRD4 inhibition-induced suppression of cell proliferation and EMT progression were mediated by caspase-1-dependent pyroptosis. Our data showed that BRD4 knockout with si-BRD4 markedly reduced cell proliferation in 786-O cells, whereas Ac-YVAD-CMK increased cell proliferation (Fig. 4a). Treatment with Ac-YVAD-CMK and JQ1 also diminished the inhibitory effect of JQ1 on the proliferation of ACHN cells (Fig. 4a).

EMT-associated proteins were also detected. Our results showed that BRD4 inhibition-elicited E-cadherin upregulation and vimentin downregulation were significantly abrogated in Ac-YVAD-CMK-treated cells (Fig. 4b, c). Furthermore, migration and invasion assays were performed, showing that Ac-YVAD-CMK ameliorated the suppression of migration and invasion caused by BRD4 deficiency or JQ1 in RCC cells (Fig. S5A–D), suggesting that BRD4 inhibition suppressed EMT progression via caspase-1-dependent pyroptosis.

In addition, BRD4 inhibition markedly increased caspase-3 activity in our results (Fig. S6A, B), indicating BRD4 deficiency induced apoptosis in RCC cells. To further analyze whether apoptosis was involved in cell proliferation and EMT progression upon BRD4 inhibition, Z-DEVD-FMK, the specific caspase-3 inhibitor, was used to block cell apoptosis. Our data showed that Z-DEVD-FMK effectively attenuated BRD4 inhibition-induced caspase-3 activation (Fig. S6A–D). However, Z-DEVD-FMK had no effect on caspase-1 (Fig. S6A–D). Similarly, BRD4 inhibition suppressed cell proliferation in RCC cells and the administration of Z-DEVD-FMK did not disturb this process (Fig. 4d). Moreover, we also employed the Edu stain measurement to see how BRD4 affected cell proliferation. As expected, Z-DEVD-FMK could not diminish the inhibitory effect of BRD4 inhibition on the proliferation of 786-O and ACHN cells (Fig. S7A, B). Thus caspase-3 played no role in BRD4 inhibition-induced suppression of cell proliferation. Furthermore, Z-DEVD-FMK could not ameliorate the suppression of EMT progression caused by BRD4 deficiency or JQ1 treatment in RCC cells (Figs. 4e, f and S5A–D). Taken together, these data revealed that caspase-1-depedent pyroptosis, but not caspase-3-dependent apoptosis, participated in BRD4 inhibition-elicited prevention of proliferation and EMT progression in RCC cells.

### JQ1 impairs tumor growth and EMT progression via pyroptosis in mice

**Fig. 5:**
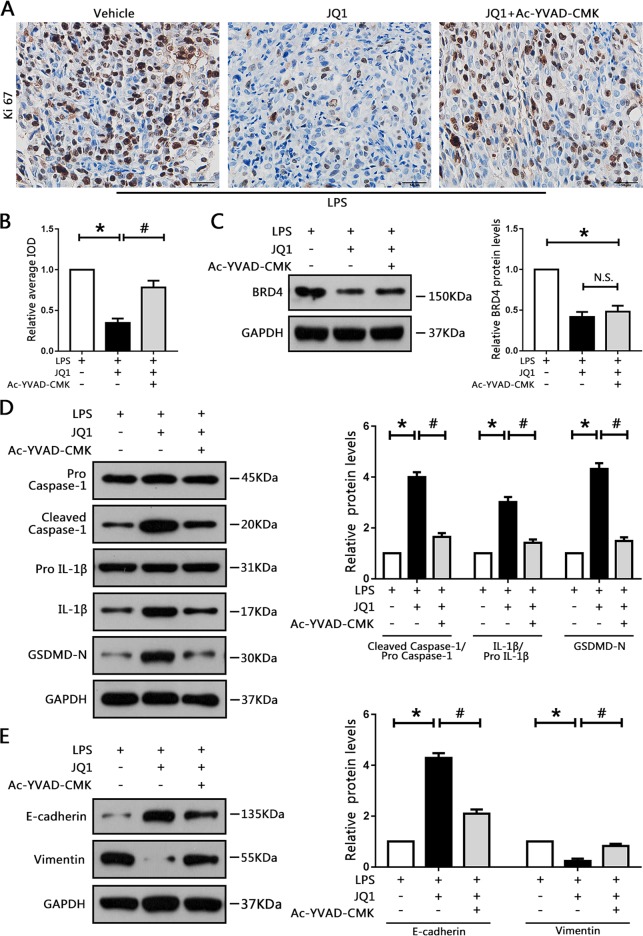
JQ1 impaired tumor growth and EMT progression via pyroptosis in mice. ACHN cells were subcutaneously inoculated into the left flanks of nude mice. Before injection, the cells were pretreated with LPS through the methods mention above, then the mice were treated with vehicle, JQ1, or combination of Ac-YVAD-CMK and JQ1. **a**, **b** Immunohistochemical staining for ki-67 in xenografted tumors. Image-Pro Plus software was used to determine the average IOD. **c**–**e** Western blot analysis of BRD4 and pyroptosis-related proteins, such as caspase-1 and IL-1β, and EMT-associated proteins, such as E-cadherin and vimentin, in xenografted specimens. *P < 0.05 vs. vehicle; ^#^*P* < 0.05 vs. JQ1; N.S. *P* > 0.05 vs. JQ1.

We then explored whether treatment with JQ1 could induce cell pyroptosis and suppress tumor growth and EMT progression in vivo. Compared with the vehicle group, JQ1-treated mice showed a strong reduction in tumor volume and weight, which could be reversed by Ac-YVAD-CMK (Fig. S8A–C). Immunohistochemical staining showed markedly decreased levels of Ki-67 in JQ1-treated mice (Fig. 5a). However, this effect could be abrogated by Ac-YVAD-CMK, as evidenced by the integral optical density (IOD) values (Fig. 5a, b). Protein levels of BRD4 were attenuated by JQ but not by Ac-YVAD-CMK (Fig. 5c). However, administration of a caspase-1 inhibitor diminished the upregulation of pyroptosis-associated proteins such as caspase-1, IL-1β and GSDMD caused by JQ1 treatment (Figs. 5d and S8D, E). Moreover, abolishing caspase-1 using Ac-YVAD-CMK reversed the JQ1-elicited decrease in vimentin levels and increase in E-cadherin levels (Fig. 5e). Collectively, these findings show that JQ1 can suppress tumor growth and EMT progression though caspase-1-dependent pyroptosis in vivo.

### BRD4 inhibition regulates NLRP3-induced pyroptosis, cell proliferation, and EMT in RCC cells

**Fig. 6:**
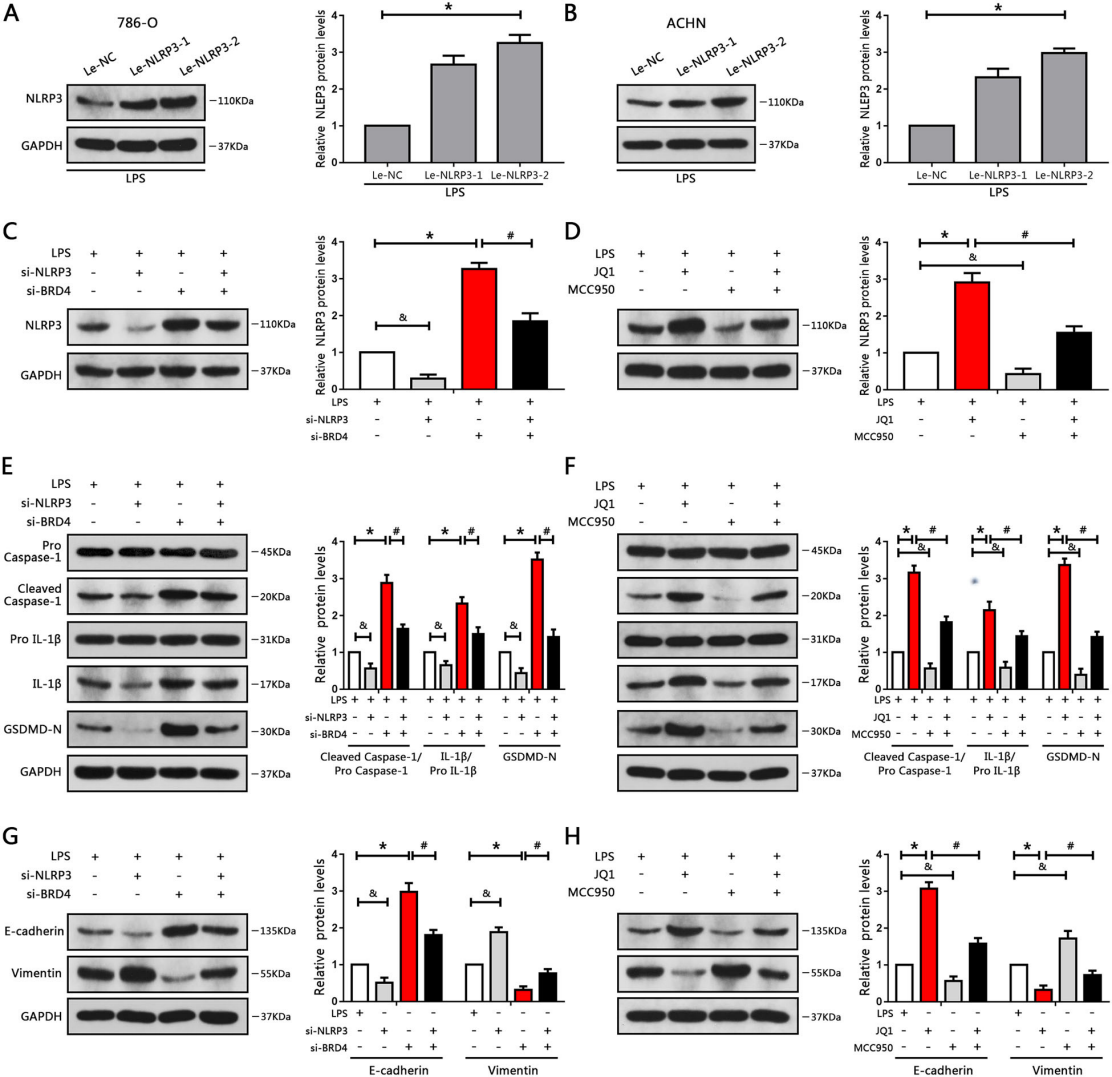
BRD4 inhibition regulated NLRP3-induced pyroptosis, cell proliferation, and EMT in RCC cells. **a**, **b** 786-O and ACHN cells were transfected with NLRP3 plasmid or control plasmid. NLRP3 levels were detected by western blotting. *P < 0.05 vs. control plasmid. **c**–**h** 786-O cells were transfected with si-NLRP3 or si-NC, then transfected with si-BRD4. ACHN cells were pre-treated with 10 μM MCC950 for 2 h, then treated with JQ1. **c**, **d** Western blot analysis of NLRP3 protein levels, and bar graphs from three independent experiments. **e**, **f** Western blot analysis of caspase-1, IL-1β and GSDMD protein levels in the indicated groups. **g**, **h** Western blot analysis of E-cadherin and Vimentin in the indicated groups. Experiments were performed in triplicate. *P < 0.05 vs. control; ^#^*P* < 0.05 vs. si-BRD4 or JQ1; ^&^*P* < 0.05 vs. si-NLRP3 or MCC950; Le-NC, control plasmid; Le-NLRP3, NLRP3 plasmid.

Previous research has demonstrated that NLRP3 triggers pyroptosis by cleavage and activation of caspase-1^30^. In this study, we enhanced the levels of NLRP3 and investigated its role in RCC cells; the results indicated that NLRP3 mRNA and protein expression levels were significantly increased after transfection with lentiviral plasmids containing NLRP3 (Figs. 6a, b and S9A). We found induction of cell pyroptosis after overexpression of NLRP3 (Figs. S9C and S10A–C), as well as inhibition of cell proliferation and EMT progression in RCC cells (Fig. S9D–G).

Next, we assessed the effects of BRD4 inhibition on NLRP3 levels. The data revealed that either si-BRD4 transfection or JQ1 treatment enhanced the levels of NLRP3 (Fig. 6c, d). To further evaluate whether BRD4 inhibition regulated pyroptosis, cell proliferation, and EMT progression though NLRP3, knockdown of NLRP3 with NLRP3-targeted siRNA or MCC950, a highly selective inhibitor of NLRP3 inflammasome, were performed. Inhibition of NLRP3 either via siRNA or MCC950 notably blocked the enhancement of NLRP3 caused by BRD4 inhibition (Figs. 6c, d and S9B). The results also showed that cell proliferation was decreased after BRD4 inhibition. However, this effect was abrogated by knockdown of NLRP3 or administration of MCC950 (Fig. S11E, F). Furthermore, blockage of NLRP3 with siRNA notably abrogated BRD4 inhibition-induced upregulation of caspase-1, IL-1β and GSDMD protein expression. Similar results were also obtained upon treatment with MCC950 and JQ1 (Fig. 6e, f). In addition, MCC950 and NLRP3 siRNA successfully blunted RCC cells pyroptosis caused by BRD4 inhibition (Fig. S10D–G). Therefore, BRD4 inhibition-induced pyroptosis and cytotoxicity in RCC cells were mediated by NLRP3 activation.

A recent study verified that upregulation of NLRP3 induced by LXRα inhibition suppressed cell metastasis, whereas NLRP3 knockout promoted migration and invasion in RCC cells^31^. Similarly, we inhibited the expression of NLRP3 and explored its role in BRD4 inhibition-suppressed EMT in RCC cells. Our results showed that BRD4 inhibition-suppressed EMT was reversed by selective knockdown of NLRP3 in 786-O cells (Fig. 6g, h).

Moreover, the results of cell wound-healing and transwell assays showed that diminishing NLRP3 levels with MCC950 blunted the suppression of migration and invasion caused by JQ1 treatment in ACHN cells (Fig. S11A–D). Collectively, these results revealed that BRD4 inhibition-elicited repression of EMT progression and proliferation and inducement of pyroptosis in RCC cells were mediated by NLRP3, and that NLRP3 might function as a tumor suppressor.

### BRD4 regulates NLRP3 expression via the NF-κB pathway

**Fig. 7:**
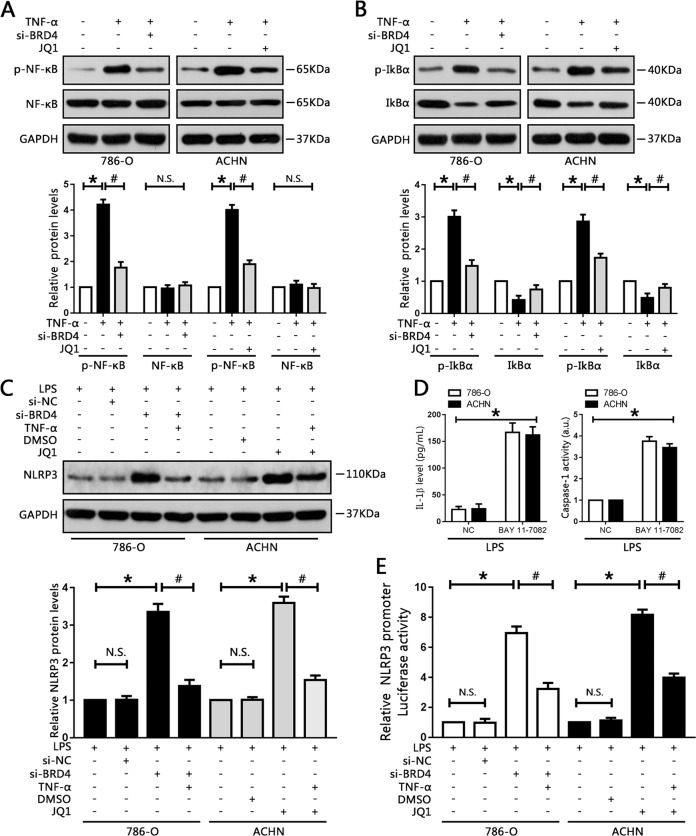
BRD4 regulated NLRP3 expression via the NF-κB pathway. 786-O and ACHN cells were pre-treated with 10 ng/ml TNF-α for 1 h before administration of si-BDR4 or JQ1, respectively. **a**, **b** Nuclear factor κB, phosphorylated NF-κB, IκBα, and phosphorylated IκBα protein levels were measured by western blotting, and quantified. *P < 0.05 vs. control. ^#^*P* < 0.05 vs. TNF-α. **c** Relative NLRP3 protein levels were measured by western blot analysis, and quantified. *P < 0.05 vs. control; ^#^*P* < 0.05 vs. si-BRD4 or JQ1; N.S. *P* > 0.05 vs. control. **d** Following treatment with 10 μM BAY 11-7082 for 1 h, levels of IL-1β secreted into the cell culture supernatants, and caspase-1 activity, were measured. *P < 0.05 vs. NC. **e** NLRP3 promoter activity in the indicated groups was measured using luciferase assay. Experiments were performed in triplicate. *P < 0.05 vs. control; ^#^*P* < 0.05 vs. si-BRD4 or JQ1; N.S. *P* > 0.05 vs. control.

To further investigate the underlying mechanism responsible for NLRP3 regulation by BRD4, possible pathways involved were explored. Recent data suggested that BET/BRD4 proteins were involved in the regulation of NF-κB in myeloproliferative neoplasms^32^. Another study showed that blockage of NF-κB exacerbated NLRP3-dependent inflammation in animal models^33^, indicating that BRD4 might be involved in the expression of NF-κB. Thus, we activated the NF-κB signaling pathway in RCC cells using TNF-α. The results showed that p-NF-κB expression was upregulated after administration of TNF-α, which could be attenuated by BRD4 knockdown in 786-O cells (Fig. 7a). Similarly, BRD4/BET inhibitor JQ1 caused significant downregulation of p-NF-κB in ACHN cells (Fig. 7a). Phosphorylation of IκBα protein levels were also obviously reduced while IκBα expression levels were moderately increased upon BRD4 inhibition (Fig. 7b), suggesting that blockage of NF-κB activation by BRD4 inhibition was mediated by suppression of IκBα phosphorylation and degradation.

Next, we investigated the role of NF-κB in the expression of NLRP3 using TNF-α, and BAY 11-7082, an inhibitor of NF-κB. Activation of NF-κB by TNF-α successfully blunted the enhancement of NLRP3 caused by BRD4 knockdown or JQ1 treatment (Fig. 7c). Moreover, there was an increase in the IL-1β secreted in the cell culture medium upon BAY 11-7082 treatment, as well as upregulation of caspase-1 activity (Fig. 7d).

Subsequently, to explore whether BRD4 could regulate NLRP3 promoter activity in RCC cells, we transfected RCC cells with a luciferase reporter plasmid containing the human NLRP3 promoter region. Our results demonstrated that inhibition of BRD4, either by siRNA or JQ1, increased NLRP3 promoter activity. However, application of TNF-α could diminish the upregulation of NLRP3 promoter activity caused by BRD4 inhibition (Fig. 7e). In summary, these findings showed that BRD4 inhibition-elicited upregulation of NLRP3 though the upstream NF-κB pathway and then transcriptionally increased the activity of the NLRP3 promoter.

### JQ1 activates NLRP3 expression and attenuates NF-κB signaling in vivo

**Fig. 8:**
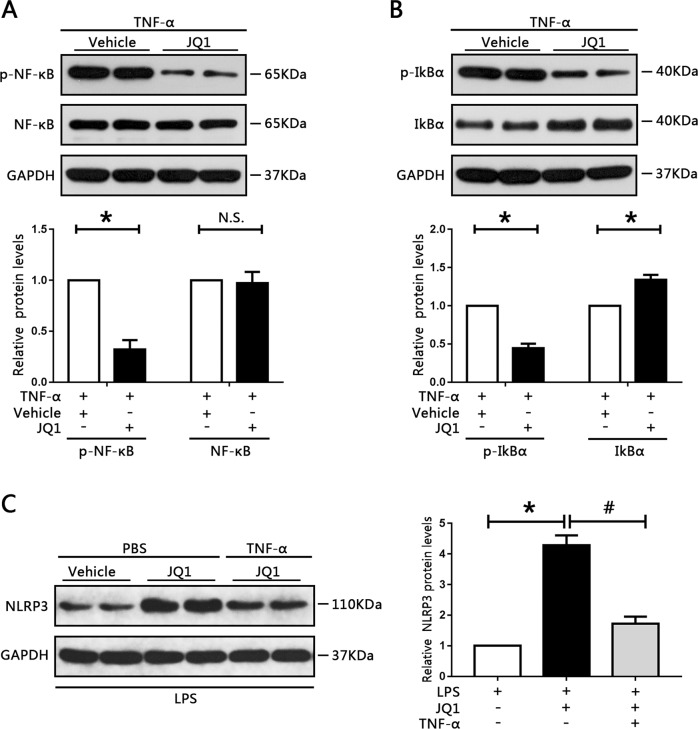
JQ1 induced NLRP3 expression and attenuated NF-κB signaling in vivo. **a** Phosphorylated NF-κB and NF-κB protein levels in RCC xenografted tumors were measured by western blotting. Data from three independent assays were quantified. **b** Western blot analysis of phosphorylated IκBα and IκBα protein levels in the indicated groups were determined and quantified. **c** The expression of NLRP3 was determined by western blotting. *P < 0.05 vs. vehicle; ^#^*P* < 0.05 vs. JQ1.

To recapitulate the findings of the in vitro studies, the effects of JQ1 in vivo were determined. NF-κB, phosphorylated NF-κB, IκBα, and phosphorylated IκBα protein levels were measured. The results showed that p-NF-κB and p-IκBα expression levels were attenuated whereas IκBα levels were increased when treated with JQ1 (Fig. 8a, b). Moreover, TNF-α could blunt the upregulation of NLRP3 levels caused by JQ1 treatment. Collectively, these results further confirm that BRD4 inhibition prevents cell proliferation and EMT progression of RCC cells through blocking NF-κB signaling and activating NLRP3-induced pyroptosis. Finally, a working model for the possible mechanism of the BRD4 inhibition-induced inhibitory effect on cell proliferation and EMT progression in RCC cells was obtained (Fig. S12).

## Discussion

Despite considerable improvements in diagnostics, adjuvant therapies, and surgical techniques for RCC patients in recent decades, patients with distant metastasis still face bleak clinical outcomes^34^. In addition, ~40% of patients with RCC exhibit metastasis or recurrence after radical resection^35^. Thus, a better understanding of molecular mechanisms in RCC and development of effective strategies is of vital importance to patients.

Accumulating evidence indicates that BRD4 is dysregulated in numerous types of cancer and influences tumor progression^36^. BRD4 has been shown to be activated in bladder cancer, where it promotes tumor progression, BRD4 knockdown negatively regulated EZH2 transcription through downregulation of C-MYC and exerted anticancer effects^37^. Suppression of BRD4 via a novel BET inhibitor decreased RCC cell proliferation and induced cell apoptosis and cycle arrest^19^. Consistent with previous studies, we found that BRD4 was upregulated in RCC tumor samples and cell lines. Inhibition of BRD4, by either genetic knockdown or JQ1, suppressed cell proliferation in RCC cells and impaired tumor growth in human RCC xenografts in mouse model. However, this effect was not seen in HK-2 cell lines, indicating that JQ1 is not toxic to normal human renal tubule epithelial cell lines. Thus, JQ1 might have therapeutic potential in patients with RCC.

EMT is a major process that promotes the escape of metastatic cancer cells from primary tumors and contributes to the aggressiveness of the tumor; during this process, cancer cells lose their epithelial characteristics, such as expression of E-ca, and gain a mesenchymal phenotype^38^. EMT has also been described as an essential event in RCC progression, metastasis, and response to therapies, and is associated with increased recurrence and decreased survival in RCC patients^39^. Inhibition of BRD4 has been shown to attenuate EMT progress in many cancer types^16,29,36^. In this study, BRD4 inhibition prevented EMT in vitro and in vivo, as evidenced by increased expression of epithelial-related proteins and downregulation of mesenchymal-associated proteins after BRD4 inhibition.

Furthermore, BRD4 has been reported to regulate the expression of tumor-related inflammatory cytokines and promote the development and progression of cancer. A study showed that JQ1 could disrupt the interactions between BRD4 and the acetylated lysine-310 residue on RelA, and inhibit the activation of TNF-α-mediated inflammatory cytokines in human lung cancer cells^40^. Deficiency of BRD4 has been reported to induce apoptosis and inhibit cell proliferation in RCC cells^19^. However, the association between BRD4 and pyroptosis in RCC remained unknown. In this study, for the first time, we revealed that pyroptosis was closely involved in the anticancer effects of BRD4 inhibition.

Inflammatory caspases cleave the gasdermin D (GSDMD) protein to trigger pyroptosis, and results in pore formation in the membrane, release of pro-inflammatory cytokines, and, finally, programed cell death^22^. Pyroptosis has long been known to occur in monocytes and macrophages; similarly, it can be triggered by chemotherapy drugs in tumor cells^20,22^. Activation of pyroptosis in cancer cells leads to suppression of cell proliferation and promotion of cell death, thereby exerting an anti-tumor effect^41^. Moreover, inhibitory effects on metastasis and cell proliferation corresponded with caspase-1-mediated pyroptosis in NSCLC^27^. Activation of pyroptosis by anthocyanin inhibited migration and invasion abilities in oral squamous cell carcinoma cells^42^. In our study, we found that caspase-1 was downregulated in RCC tissues and cell lines. Importantly, BRD4 inhibition induced pyroptosis by increasing caspase-1, IL-1β and GSDMD levels in vitro and vivo. We further found that caspase-1 inhibitor Ac-YVAD-CMK ameliorated the activation of pyroptosis induced by BRD4 knockout. Moreover, the inhibitory effects of JQ1 on cell proliferation and EMT were attenuated by Ac-YVAD-CMK, further demonstrating that pyroptosis has a crucial function in BRD4 inhibition-mediated cell proliferation and EMT. Furthermore, it was found that BRD4 inhibition also increased the levels of caspase-3 in RCC cells. We then used Z-DVED-FMK to block apoptosis on RCC cells and verified that caspase-3 played no role in BRD4 inhibition-induced suppression of cell proliferation. Therefore, the antitumor effects of BRD4 inhibition were due to caspase-1-mediated pyroptosis, but not caspase-3-dependent apoptosis. However, the association between apoptosis and pyroptosis induced by BRD4 inhibition in RCC cells will require further investigation.

NLRP3 belongs to the nucleotide-binding domain and leucine-rich repeat-containing protein family, which respond to a variety of infections and endogenous ligands. It is involved in the formation of the NLRP3 inflammasome that activates caspase-1, initiating the inflammatory form of programmed cell death known as pyroptosis^43^. Accumulating evidence suggests that the NLRP3 inflammasome influences the pathogenesis of cancer by regulating immune responses, cell proliferation, and death^44^. NLRP3 activity is associated with increased lung metastasis; mice lacking NLRP3 showed reduced tumor burden and lung metastasis compared with those expressing NLRP3 normally^45^. However, other studies have shown that mice lacking NLRP3 are more susceptible to colitis-related colorectal cancer, indicating that the NLRP3 inflammasome has an antitumor role in colitis-associated cancer^46^. Moreover, NLRP3 levels were low in RCC tumor samples, and that it might function as a tumor suppressor in RCC^31^. Consistent with this, our study showed that BRD4 deficiency markedly increased NLRP3 protein expression and enhanced its transcriptional activity, and also led to suppression of cell proliferation and EMT and induction of pyroptosis. Furthermore, we inhibited NLRP3 expression with targeted siRNA or a pharmaceutical inhibitor, the promotion of pyroptosis and inhibition of cell proliferation and EMT caused by JQ1 treatment or BRD4 inhibition were weakened. Thus, we revealed that BRD4 inhibition exerted an anticancer effect through inducing NLRP3 inflammasomes-regulated pyroptosis.

NF-κB plays a crucial part in the regulation of genes involved in immunity and inflammation^47^. In cancer cells, NF-κB promotes an invasive phenotype and transcription of oncogenes^48^. Activation of NF-κB has also been observed in various types of cancer, including RCC^49^. Studies have shown that BET/BRD4 proteins are involved in the regulation of NF-κB in a variety of cancers^32^. Moreover, JQ1 disrupted the interactions between BRD4 and NF-κB, and blocked the activation of NF-κB signaling in cancer cells^32,40^. Based on these findings, we investigated the possible regulators of NLRP3 after BRD4 inhibition in RCC and surmised that NF-κB signaling was involved in this process. Furthermore, the NF-κB signaling pathway is closely associated with NLRP3. On the one hand, NF-κB binds to the NLRP3 promoter region and affects transcriptional regulation of NLRP3^50^. On the other hand, blockage of NF-κB exacerbates the activation of the NLRP3-dependent inflammasome^33^. Consistent with this, we demonstrated that knockout of BRD4 with siRNA decreased the phosphorylation of NF-κB and subsequently activated the downstream NLRP3 pathway. In addition, a combination of JQ1 and TNF-α, resulted in a reduction in NLRP3 levels. Moreover, either BRD4 siRNA or JQ1 increased the activity of the NLRP3 promoter, and application of TNF-α diminished this enhancement. Therefore, these findings demonstrate that BRD4 inhibition elicits enhancement of NLRP3 expression though the upstream NF-κB pathway and then transcriptionally increases the activity of the NLRP3 promoter. In addition, BRD4 exerts many of its physiological effects on cells and regulates many gene expression^11^. Although we revealed that BRD4 inhibition could induce pyroptosis by activating NLRP3 inflammasome, other mechanism for activation of pyroptosis by BRD4 inhibition needs to be further investigated. Moreover, inflammation triggered by inflammasomes results in promotion of anti-tumor immunity conferred by NK cells and T cells that is detrimental to the development of cancer^44^, whether this effect contribute to the proliferation and EMT progression will requires further investigation.

In summary, our study demonstrated the anti-tumor effect of a BRD4 inhibitor in RCC. Further, we identified that BRD4 inhibition prevented cell proliferation and EMT progression. This effect may be attributable, at least in part, to the activation of NLRP3 inflammasome-induced pyroptosis through NF-κB signaling. Thus, our results indicate that BRD4 is a promising target for RCC therapy.

## Materials and methods

### Cell lines and reagents

Five renal cell carcinoma cell lines (786-O, ACHN, A498, CAKI-1, and OSRC-2) and a human renal tubule epithelial cell line (HK-2) were purchased from the American Type Culture Collection (Manassas, USA). 786-O and OSRC-2 cell lines were maintained in RPMI-1640 culture medium (GIBCO), ACHN and A498 cell lines were maintained in minimal essential medium with Earle’s balanced salts, and CAKI‑1 cells were cultured in McCoy’s 5A medium. All culture media were supplemented with 10% heat-inactivated FBS (FBS; GIBCO) and 1% streptomycin–penicillin. HK-2 cell line was cultured in complete medium containing K-SFM (keratinocyte serum-free medium). All cell lines were maintained in an incubator with 5% CO_2_ at 37 °C. JQ1 (Catalog NO. S7110), MCC950 (Catalog NO. S7809), and BAY 11-7082 (Catalog NO. S2913), Z-DEVD-FMK (Catalog NO. S7312) were all purchased from Selleckchem. Ac-YVAD-CMK (Catalog NO. N-1330) was purchased form BaChem, and TNF-α (Catalog NO. 300-01A) was obtained from PeproTech.

### Patient samples

This study had been carried out in accordance with the World Medical Association Declaration of Helsinki, and that all subjects provided written informed consent. A total of 40 RCC specimens with paired normal tissue samples (1 cm away from the margin of the tumor tissues) were collected from patients at the urology department of Renmin Hospital of Wuhan University from 2016 to 2018. None of the patients had received chemotherapy or radiotherapy prior to the operation. Histological and pathological diagnoses were evaluated by two senior pathologists. This experiment was approved by the Institutional Ethics Committee of Renmin Hospital of Wuhan University and strictly followed the guidelines of the Institutional Ethics Committee. Informed consent was obtained from patients before the operation.

### Pyroptosis and inflammasome activation

To activate pyroptosis and inflammasome, RCC cells were pretreated with lipopolysaccharide (LPS) (0.5 μg/ml) (Sigma, USA) for 4 h, then incubated with ATP (5 nM) for 1 h in complete medium, as previously described^31^.

### Immunohistochemistry and Histology

Tissues were fixed in 4% paraformaldehyde, embedded in paraffin, and then used to prepare 4.0 μm sections. Subsequently, sections were deparaffinized, rehydrated, and incubated with primary antibodies overnight at 4 °C. After incubation, the sections were washed three times with phosphate-buffered saline (PBS) and incubated with HRP (horseradish peroxidase)-conjugated secondary antibody (Servicebio, China). Finally, all sections were stained with DAB (3,3-diaminobenzidine, Servicebio, China) and visualized using a light microscope (Olympus, Japan). The primary antibodies used for immunohistochemistry were anti-KI67 (ab15580, Abcam), anti-BRD4 (ab128874; Abcam), anti-caspase-1 (ab1872; Abcam), and anti-Il-1β (ab7632; Abcam). For quantitation, the average IOD was determined using Image-Pro Plus software (Version 6.0). The relative mean IOD of each group was divided by that of the control group. For histological staining, specimens were stained with hematoxylin and eosin (H&E) to evaluate nucleus and cytoplasm.

### Quantitative real-time PCR

Total RNA from tissues and cell lines was extracted with TRIzol regent (Invitrogen, USA). The concentration and purity of RNA was measured by spectrophotometry, and complementary DNA was synthesized using the PrimeScript RT Reverse transcriptase reagent kit (Takara) following the manufacturer’s protocol. The qRT-PCR analysis was performed using s SYBR Premix Ex Taq kit (Takara) and the StepOnePlus Real-Time PCR System (Applied Biosysterms, USA). All results were normalized to GAPDH, and the ΔΔCt method was used to determine the relative expression of genes. The target gene primers were designed by Shanghai Sangon Biotech Co., Ltd (Shanghai, China) and are listed in Supplemental Table 1.

### Cell proliferation assay

RCC cells were seeded in a 96-well plate at a density of 5 × 10^3^ cells/100 μL per well and incubated overnight. After transfection with BRD4 siRNA or si-NC, they were treated with JQ1 or DMSO, respectively. CCK8 reagent (10 μL) (Dojindo, Japan) was added to each well. Then, cells were incubated at room temperature in the dark for 2 h. The absorbance value was measured using a spectrophotometer at 450 nm. GraphPad Prism 7 was used to measure IC_50_ values (GraphPad Software, Inc.).

### Plasmids and RNA interference

siRNAs targeting different sequences of BRD4 and NLRP3, and negative controls were designed and synthesized by GenePharma Company (Shanghai, China). For knockdown experiments, 786-O cells (density of 2 × 10^5^ cells per well) were cultured in six-well plates and transfected with siRNA targeted to BRD4 or NLRP3 using a Lipofectamine 2000 kit, according to the manufacturer’s instructions. After 6 incubation, the medium was replaced and cells were incubated for an additional 42 h. NLRP3 overexpression lentiviral plasmids or control lentiviral plasmids were designed and constructed by GenePharma Company (Shanghai, China). For the overexpression assay, 786-O and ACHN cells were infected with lentiviral plasmids for 24 h, then the medium was exchanged and cells were cultured for an additional 24 h.

### Colony formation

786-O and ACHN cells were plated onto a six-well plate. After attachment, the cells were transfected with BRD4 siRNA or si-NC or were treated with JQ1 or DMSO, respectively. Then, the cells were harvested using 0.25% trypsin and resuspended in culture medium. Subsequently, 1000 cells/per well were seeded in six-well plates and cultured in drug-free complete culture medium for 10 days. Finally, the colonies were fixed with 4% paraformaldehyde, and crystal violet solution was used to stain the colonies (Beyotime, China). The number of positive colonies (>50 cells/colony) were counted under a microscope (Olympus, Japan); results were expressed as mean ± SD of three independent experiments.

### Cell wound-healing

In this study, 786-O and ACHN cells were seeded into a six-well plate and incubated in medium containing 10% FBS overnight. Then, cells were transfected with BRD4 siRNA or si-NC or were treated with JQ1 or DMSO respectively. The wound was scraped using a 200 μL pipette tip and washed with PBS to remove cell debris. Subsequently, the supernatant was replaced with serum-free medium. Scratch gaps were photographed at 0 and 24 h using a microscope (magnification, x200; Olympus, Japan).

### Cell invasion

In this study, 786-O and ACHN cells were seeded into a six-well plate, followed by BRD4 siRNA or si-NC transfection, and JQ1 or DMSO treatment, respectively, for 48 h. Cells were harvested with 0.25% trypsin and resuspended in serum-free medium. For the cell invasion assays, 1 × 10^5^ cells/200 μL per well were seeded in a transwell chamber pretreated coated with Matrigel in the upper chamber. 500 μL complete medium (containing 10% FBS) was placed in the lower chamber as an inducer. Twenty four hours later, a cotton swab was used to remove cells located in the upper chamber; cells on the lower side of the chamber were fixed with 4% paraformaldehyde (Beyotime, China) and stained with 0.5% crystal violet solution. Finally, the cells were counted and photographed using a microscope (Olympus, Japan).

### ELISA analysis

The production of IL-1β in the culture supernatant was detected with ELISA kits (Beyotime, China), following the instructions of the manufacturer.

### Flow cytometry assay

FAM-FLICA Caspase-1 Detection Kit was used to determine pyroptosis, according to the manufacturer’s instructions (ImmunoChemistry, USA). Cells were transfected with si-BRD4 or treated with JQ1, the samples were harvested and stained with PI and FAM-YVAD-FMK (a caspase-1 specific marker), then flow cytometer (BD FACSalibur, USA) was used to detect the samples, FAM-YVAD-FMK and PI double stained cells were defined as pyroptosis.

### Measurement of caspase-1 activity

Caspase-Glo® 1 inflammasome assays (Promega, USA) were performed to evaluate caspase-1 activity, following the instructions of the manufacturer. Briefly, cells were seeded in 96-well plates and Caspase-Glo 1 reagent was added to each well. Then, cells were incubated at room temperature for 1 h. Subsequently, cells were shaken for 5 min and incubated again at room temperature for 30 min. Victor X (PerkinElmer, USA) was used to detect luminescence.

### 5-Ethynyl-2′-deoxyuridine assay

Cell-Light 5-Ethynyl-2′-deoxyuridine (EdU) imaging detection kit was used to analyze cell growth, following the instructions of the manufacturer (RiboBio, China). Briefly, cells were cultured with 10 µM Edu in medium, then fixed with 4% paraformaldehyde (Beyotime, China) and stained with 5 µg/ml Hoechst 33342. Finally, Edu and Hoechst positive cells were counted and photographed using a microscope (magnification, ×200; Olympus, Japan).

### Western blotting analysis

Cells and tissues were collected and lysed with RIPA buffer containing phenylmethanesulfonylfluoride (Beyotime, China). Protein concentration was determined with a BCA assay kit (Beyotime, China). Equivalent amounts of total protein were separated by 10% sodium dodecyl sulfate polyacrylamide (servicebio, China) gel electrophoresis and then transferred to polyvinylidene fluoride membranes (servicebio, China). Blocking was performed in TBST containing 5% nonfat milk, and membranes were incubated with primary antibody overnight at 4 °C. Subsequently, membranes were incubated with HRP-conjugated secondary antibodies for 1 h at 37 °C. An ECL kit (Multisciences, China) was used to detect the proteins. Image J software (NIH, MD, USA) was used to evaluate the relative expression levels of different proteins. Primary antibodies for BRD4 (ab128874), caspase-1 (ab1872), Il-1β (ab7632), cleaved N-terminal GSDMD (ab215203), vimentin (ab92547), caspase-3 (ab2302) and GAPDH (ab9485) were purchased from Abcam. Antibodies for Cl-caspase-1 (4199), E-ca (3195), NLRP3 (15101), NF-κB-p65 (8242), phospho-NF-κB-p65 (3033), IkBa (4812), and p-IkBa (2859) were from Cell Signaling Technology. Goat and anti-rabbit secondary antibodies were purchased from Wuhan Boster Bio-engineering Limited Company, China.

### Luciferase reporter assay

The NLRP3 promoter reporter vector was designed and constructed by Shanghai Sangon Biotech Co., Ltd (Shanghai, China). The 786-O cells were transiently transfected with luciferase reporter plasmids, Renila control luciferase vector, and si-BRD4 or si-NC using Lipofectamine 2000 transfection reagent, following the protocols provided by the manufacturer. For the ACHN group, cells were transfected with luciferase reporter plasmids, Renila control luciferase vector, and JQ1 or DMSO. After 48 h, cells were collected and lysed. A Dual Luciferase Reporter Assay System (Promega, USA) was used to measure luciferase activity. Experiments were performed three times with six replicates for each condition, and results were normalized to the Renila luciferase vector.

### Animal assay

Animal experiments were approved by the Animal Ethics Committee of Wuhan University. All 4–5-week-old BALB/C nude mice (Beijing Vital River Laboratory Animal, China) were housed under pathogen-free conditions. ACHN cells were prepared by suspending 5 × 10^6^ cells in 100 µL PBS and subcutaneously injected into the left flank of nude mice. Before injection, the pyroptosis and inflammasome in RCC cells were activated through the methods mention above. When the tumors were of palpable size (100 mm^3^), the mice were randomized into various groups. Each group contained five mice. JQ1 in DMSO was diluted by dropwise addition of physiological saline to obtain a final concentration of 5 mg/ml. For the JQ1 treatment groups, mice were fasted for 2 h prior to intraperitoneal treatment with diluted JQ1 (50 mg/kg/day) at day 8. For the control group, mice were treated with an equal volume of physiological saline containing 5% DMSO. For the JQ1 + Ac-YVAD-CMK group, mice were fasted for 2 h prior to administration of JQ1, then fed by oral gavage with caspase-1 inhibitor Ac-YVAD-CMK (0.1 mg/kg/day).

To further explore whether JQ1 could activate regulated NLRP3 expression via the NF-κB pathway in vivo, tumor xenografting in nude mice was performed. For the TNF-α group, a total of 2 μg TNF-α was injected directly into tumors each day. Subsequently, mice received intraperitoneal treatment with diluted JQ1. For the PBS group, an equal volume of PBS was injected directly into tumors each day, after which mice received intraperitoneal treatment with diluted JQ1 or the vehicle. Tumor volume was evaluated every 5 days with Vernier calipers and calculated using the equation: 0.5 × length × width^2^ (mm^3^). After 30 days, all mice were sacrificed.

### Statistical analysis

Statistical analysis was conducted using SPSS 20.0 software (SPSS Inc, USA). All experiments were performed at least three times; data are presented as mean ± SD. Student’s *t*-test and analysis of variance was used to evaluate statistically significant differences. The Bonferroni post hoc test was used for multiple comparisons. Statistical significance was set as *P* < 0.05.

## Supplementary information

Supplementary Figure legends

Supplementary Figure 1

Supplementary Figure 2

Supplementary Figure 3

Supplementary Figure 4

Supplementary Figure 5

Supplementary Figure 6

Supplementary Figure 7

Supplementary Figure 8

Supplementary Figure 9

Supplementary Figure 10

Supplementary Figure 11

Supplementary Figure 12

Supplementary Table legends

Supplementary Table 1

## Data Availability

All of the data in this paper are available from the corresponding author on reasonable request.

## References

[1] Ferlay J (2015). Cancer incidence and mortality worldwide: sources, methods and major patterns in GLOBOCAN 2012. Int. J. Cancer.

[2] Jonasch E, Gao J, Rathmell WK (2014). Renal cell carcinoma. BMJ.

[3] Siegel RL, Miller KD, Jemal A (2019). Cancer statistics, 2019. CA Cancer J. Clin..

[4] Ljungberg B (2011). Corrigendum to “The Epidemiology of Renal Cell Carcinoma” [Eur Urol 2011;60:615-21]. Eur. Urol..

[5] Scelo G (2017). Genome-wide association study identifies multiple risk loci for renal cell carcinoma. Nat. Commun..

[6] Ljungberg B (2015). EAU guidelines on renal cell carcinoma: 2014 update. Eur. Urol..

[7] Barata PC, Rini BI (2017). Treatment of renal cell carcinoma: current status and future directions. CA Cancer J. Clin..

[8] Jung M, Gelato KA, Fernandez-Montalvan A, Siegel S, Haendler B (2015). Targeting BET bromodomains for cancer treatment. Epigenomics-Uk.

[9] Liu Z (2017). Drug discovery targeting bromodomain-containing protein 4. J. Med. Chem..

[10] Leal AS (2017). Bromodomain inhibitors, JQ1 and I-BET 762, as potential therapies for pancreatic cancer. Cancer Lett..

[11] Ferri E, Petosa C, McKenna CE (2016). Bromodomains: structure, function and pharmacology of inhibition. Biochem. Pharmacol..

[12] Filippakopoulos P, Knapp S (2014). Targeting bromodomains: epigenetic readers of lysine acetylation. Nat. Rev. Drug Discov..

[13] Zaware N, Zhou MM (2017). Chemical modulators for epigenome reader domains as emerging epigenetic therapies for cancer and inflammation. Curr. Opin. Chem. Biol..

[14] Doroshow DB, Eder JP, LoRusso PM (2017). BET inhibitors: a novel epigenetic approach. Ann. Oncol..

[15] Wang B (2018). FBP1 loss contributes to BET inhibitors resistance by undermining c-Myc expression in pancreatic ductal adenocarcinoma. J. Exp. Clin. Cancer Res.

[16] Tan Y (2018). Inhibition of BRD4 suppresses tumor growth in prostate cancer via the enhancement of FOXO1 expression. Int. J. Oncol..

[17] Huang M (2017). The suppression of bromodomain and extra-terminal domain inhibits vascular inflammation by blocking NF-kappaB and MAPK activation. Br. J. Pharm..

[18] Wang J (2019). BRD4 inhibition attenuates inflammatory response in microglia and facilitates recovery after spinal cord injury in rats. J. Cell. Mol. Med..

[19] Chen W (2018). Development and evaluation of a novel series of Nitroxoline-derived BET inhibitors with antitumor activity in renal cell carcinoma. Oncogenesis.

[20] Wang Y (2017). Chemotherapy drugs induce pyroptosis through caspase-3 cleavage of a gasdermin. Nature.

[21] Xi H (2016). Caspase-1 inflammasome activation mediates homocysteine-induced pyrop-apoptosis in endothelial cells. Circ. Res..

[22] Yuan J, Najafov A, Py BF (2016). Roles of caspases in necrotic cell death. Cell.

[23] Xue M (2019). Empagliflozin prevents cardiomyopathy via sGC-cGMP-PKG pathway in type 2 diabetes mice. Clin. Sci. (Lond.).

[24] Kayagaki N (2011). Non-canonical inflammasome activation targets caspase-11. Nature.

[25] Wree A (2014). NLRP3 inflammasome activation results in hepatocyte pyroptosis, liver inflammation, and fibrosis in mice. Hepatology.

[26] Li X (2014). MicroRNA-30d regulates cardiomyocyte pyroptosis by directly targeting foxo3a in diabetic cardiomyopathy. Cell Death Dis..

[27] Wang F (2018). Simvastatin suppresses proliferation and migration in non-small cell lung cancer via pyroptosis. Int J. Biol. Sci..

[28] Chu Q (2016). Pyroptosis is involved in the pathogenesis of human hepatocellular carcinoma. Oncotarget.

[29] Hu X (2017). Prolyl isomerase PIN1 regulates the stability, transcriptional activity and oncogenic potential of BRD4. Oncogene.

[30] Hughes MM, O’Neill L (2018). Metabolic regulation of NLRP3. Immunol. Rev..

[31] Wang K (2019). LXRalpha promotes cell metastasis by regulating the NLRP3 inflammasome in renal cell carcinoma. Cell Death Dis..

[32] Kleppe M (2018). Dual targeting of oncogenic activation and inflammatory signaling increases therapeutic efficacy in myeloproliferative neoplasms. Cancer Cell.

[33] Afonina IS, Zhong Z, Karin M, Beyaert R (2017). Limiting inflammation-the negative regulation of NF-kappaB and the NLRP3 inflammasome. Nat. Immunol..

[34] Patard JJ (2005). Prognostic value of histologic subtypes in renal cell carcinoma: a multicenter experience. J. Clin. Oncol..

[35] Ko JJ (2015). The International Metastatic Renal Cell Carcinoma Database Consortium model as a prognostic tool in patients with metastatic renal cell carcinoma previously treated with first-line targeted therapy: a population-based study. Lancet Oncol..

[36] Andrieu G, Tran AH, Strissel KJ, Denis GV (2016). BRD4 regulates breast cancer dissemination through Jagged1/Notch1 signaling. Cancer Res..

[37] Wu X (2016). BRD4 regulates EZH2 transcription through upregulation of C-MYC and represents a novel therapeutic target in bladder cancer. Mol. Cancer Ther..

[38] Lambert AW, Pattabiraman DR, Weinberg RA (2017). Emerging biological principles of metastasis. Cell.

[39] Piva F (2016). Epithelial to mesenchymal transition in renal cell carcinoma: implications for cancer therapy. Mol. Diagn. Ther..

[40] Zou Z (2014). Brd4 maintains constitutively active NF-kappaB in cancer cells by binding to acetylated RelA. Oncogene.

[41] Zhu M (2018). Design, synthesis, and evaluation of chalcone analogues incorporate alpha,beta-Unsaturated ketone functionality as anti-lung cancer agents via evoking ROS to induce pyroptosis. Eur. J. Med. Chem..

[42] Yue E (2019). Anthocyanin is involved in the activation of pyroptosis in oral squamous cell carcinoma. Phytomedicine.

[43] Bruchard M (2015). The receptor NLRP3 is a transcriptional regulator of TH2 differentiation. Nat. Immunol..

[44] Karki R, Man SM, Kanneganti TD (2017). Inflammasomes and cancer. Cancer Immunol. Res..

[45] Chow MT (2012). NLRP3 suppresses NK cell-mediated responses to carcinogen-induced tumors and metastases. Cancer Res..

[46] Allen IC (2010). The NLRP3 inflammasome functions as a negative regulator of tumorigenesis during colitis-associated cancer. J. Exp. Med..

[47] Sun SC (2017). The non-canonical NF-kappaB pathway in immunity and inflammation. Nat. Rev. Immunol..

[48] Shostak K, Chariot A (2015). EGFR and NF-kappaB: partners in cancer. Trends Mol. Med..

[49] Ikegami A (2018). Knockdown of NF-kappaB1 by shRNA Inhibits the Growth of Renal Cell Carcinoma In Vitro and In Vivo. Oncol. Res..

[50] Qiao Y, Wang P, Qi J, Zhang L, Gao C (2012). TLR-induced NF-kappaB activation regulates NLRP3 expression in murine macrophages. Febs Lett..

