# Supplementary material for: Inhibition of BRD4 prevents proliferation and epithelial–mesenchymal transition in renal cell carcinoma via NLRP3 inflammasome-induced pyroptosis
Source: Cell Death Dis. 2020 Apr 17;11(4):239. doi: 10.1038/s41419-020-2431-2 (PMC7165180; doi:10.1038/s41419-020-2431-2)
Supplement: Supplementary file 1 — Supplementary Figure legends [file 41419_2020_2431_MOESM1_ESM.doc]

**Supplementary figure 1: Upregulation of BRD4 in RCC tissue specimens and cell lines.**

(A) Immunohistochemical analysis of BRD4 in RCC specimens and adjacent normal renal tissues. (B) Real-time qPCR analysis of BRD4 mRNA levels in HK-2 cells and five renal cell carcinoma cell lines (A498, ACHN, CAKI-1, 786-O, and OSRC-2). Data are represented as means ± SD. *P < 0.05, relative to HK-2 cells.

**Supplementary figure 2: Suppression of cell proliferation by BRD4 inhibition in RCC cell lines.**

(A, B) 786-O cells were transfected with siBRD4, and the transfection efficiency was confirmed using RT-qPCR and western blotting. (C, D) Cell proliferation of HK-2 cells following treatment with various concentrations of JQ1 for different durations was evaluated using CCK8 assay. (E-H) Cell proliferation of RCC cells following treatment with with JQ1 (1.92 nM, 3.2 nM, 9.6 nM, 16 nM, 48 nM, 80 nM, 240 nM, 400 nM, 1.2 μM, 2 μM, 6 μM, 10 μM, 30 μM, and 50 μM) for 48 hours was evaluated using CCK8 assay, and the IC_50_ values for each cell line were calculated. (I–L) Cell proliferation of RCC cells following treatment with various concentrations of JQ1 for 0, 24, and 48 hours was evaluated using CCK8 assay. *P < 0.05 relative to the DMSO group.

**Supplementary figure 3: Suppression of RCC cell invasion and migration via inhibition of BRD4.**

(A, B) Transwell assay was used to determine cell invasive ability in 786-O and ACHN cells following transfection with siBRD4 or treatment with JQ1. The number of cells was calculated in three random fields at 200X magnification. *P < 0.05 vs. si-NC or DMSO. (C, D) Wound healing assay was used to determine 786-O and ACHN cell migratory ability following transfection with siBRD4 or treatment with JQ1.

**Supplementary figure 4: BRD4 inhibition activated caspase-1-dependent pyroptosis *in vitro*.**

(A) Flow cytometry assay was used to determine cell pyroptosis in 786-O and ACHN cells following transfection with siBRD4 or treatment with JQ1. (B) 786-O and ACHN cells were pre-treated with 50 μM Ac-YVAD-CMK for 24h, then transfected with siBRD4 or treated with JQ1, respectively. Flow cytometry assay was used to determine cell pyroptosis. The bar graph shows the percent of FAM-YVAD-FMK and PI double stained cells from three independent experiments. *P < 0.05 vs. control; #P < 0.05 vs. si-BRD4 or JQ1

**Supplementary figure 5: Inhibition of BRD4 suppressed cell metastasis via pyroptosis *in vitro*.**

786-O cells were pre-treated with 50 μM Ac-YVAD-CMK or 50 μM Z-DEVD-FMK for 24 h, then transfected with siBRD4; ACHN cells were pre-treated with 50 μM Ac-YVAD-CMK or 50 μM Z-DEVD-FMK for 24 h, then treated with JQ1. (A, B) Cell invasive ability was measured using the transwell assay. *P < 0.05 vs. control; #P < 0.05 vs. siBRD4 or JQ1; N.S. P > 0.05 vs. si-BRD4 or JQ1. (C, D) Cell migratory ability was measured using the wound healing assay.

**Supplementary figure 6: BRD4 inhibition activated apoptosis *in vitro*.**

786-O cells were pre-treated with 50 μM Ac-YVAD-CMK or 50 μM Z-DEVD-FMK for 24 h, then transfected with siBRD4; ACHN cells were pre-treated with 50 μM Ac-YVAD-CMK or 50 μM Z-DEVD-FMK for 24 h, then treated with JQ1. (A, B) Activity of caspase-3 and caspase-1 were measured. (C, D) Western blots of caspase-3 and caspase-1 levels in 786-O and ACHN cells. The bar graph shows the relative levels of caspase-3 and caspase-1 from three independent experiments. *P < 0.05 vs. control; #P < 0.05 vs. si-BRD4 or JQ1; N.S. P > 0.05 vs. si-BRD4 or JQ1

**Supplementary figure 7: Pyroptosis activation mediated BRD4 inhibition-induced suppression of cell proliferation.**

786-O cells were pre-treated with 50 μM Ac-YVAD-CMK or 50 μM Z-DEVD-FMK for 24 h, then transfected with siBRD4; ACHN cells were pre-treated with 50 μM Ac-YVAD-CMK or 50 μM Z-DEVD-FMK for 24 h, then treated with JQ1. (A, B) Edu staining was used to determine cell proliferation. The number of Edu and Hoechst positive cells were counted in three random fields at 200X magnification. *P < 0.05 vs. control; #P < 0.05 vs. si-BRD4 or JQ1; N.S. P > 0.05 vs. si-BRD4 or JQ1.

**Supplementary figure 8. JQ1 impaired tumor growth via pyroptosis in mice.**

ACHN cells were subcutaneously inoculated into the left flanks of nude mice, then the mice were treated with vehicle, JQ1, or a combination of caspase-1 inhibitor and JQ1. (A) Image of tumors obtained from mice. (B, C) Tumor weights were measured and a volume curve was generated. (D, E) Immunohistochemical analysis of caspase-1 and IL-1β in xenografted tissues. Image-Pro Plus software was used to analyze the average IOD. *P < 0.05 vs. vehicle; #P < 0.05 vs. JQ1.

**Supplementary figure 9: Overexpression of NLRP3 activated cell pyroptosis and suppressed cell proliferation and metastasis.**

(A) 786-O and ACHN cells were transfected with lentiviral plasmid containing NLRP3 or control plasmid. The transfection efficiency was confirmed using RT-qPCR. *P < 0.05 vs. control plasmid. (B) 786-O cells were transfected with si-NLRP3 or si-NC, then transfected with si-BRD4. NLRP3 levels were detected by RT-qPCR. *P < 0.05 vs. control; #P < 0.05 vs. si-BRD4; &P < 0.05 vs. si-NLRP3. (C-G) 786-O and ACHN cells were transfected with plasmid NLRP3 plasmid or control plasmid (C) Pyroptosis-related proteins caspase-1, IL-1β and GSDMD were detected by western blotting. (D) Vimentin and E-cadherin levels were measured by western blotting. (E) Cell proliferation was evaluated using CCK8 assay. (F) Cell invasive ability was measured using the transwell assay. (G) Cell migratory ability was measured using the wound healing assay. *P < 0.05 vs. control plasmid. Le-NC, control plasmid; Le-NLRP3, NLRP3 plasmid.

**Supplementary figure 10: BRD4 inhibition activated NLRP3-induced pyroptosis in RCC cells.**

(A-C) Flow cytometry assay was used to determine cell pyroptosis in 786-O and ACHN cells following transfection with NLRP3 plasmid or control plasmid. *P < 0.05 vs. control plasmid. (D-G) 786-O cells were transfected with si-NLRP3 or si-NC, then transfected with si-BRD4. ACHN cells were pre-treated with 10 μM MCC950 for 2 h, then treated with JQ1. The bar graph shows the percent of FAM-YVAD-FMK and PI double stained cells from three independent experiments. *P < 0.05 vs. control; #P < 0.05 vs. si-BRD4 or JQ1; &P < 0.05 vs. si-NLRP3 or MCC950.

**Supplementary figure 11: Inhibition of BRD4 suppressed cell proliferation and metastasis via NLRP3 in RCC cells**.

(A) 786-O cells were transfected with si-NLRP3 or si-NC, then transfected with si-BRD4. ACHN cells were pre-treated with or without MCC950 for 2h, then treated with JQ1. (A, B) Transwell assay analysis of cell invasion in the indicated groups. Each experiment was performed in triplicate. (C, D) Cell migratory ability was measured using the wound healing assay. (E, F) Cell proliferation in the indicated groups was evaluated using the CCK8 assay. *P < 0.05 vs. control; #P < 0.05 vs. si-BRD4 or JQ1; &P < 0.05 vs. si-NLRP3 or MCC950.

**Supplementary figure 12:** A mechanistic model of JQ1-induced inhibition of cell proliferation and EMT progression through activation of NLRP3-induced pyroptosis via inhibition of NF-κB signaling in RCC cells.
