# Supplementary figures and images for: Inhibition of BRD4 prevents proliferation and epithelial–mesenchymal transition in renal cell carcinoma via NLRP3 inflammasome-induced pyroptosis
Source: Cell Death Dis. 2020 Apr 17;11(4):239. doi: 10.1038/s41419-020-2431-2 (PMC7165180; doi:10.1038/s41419-020-2431-2)

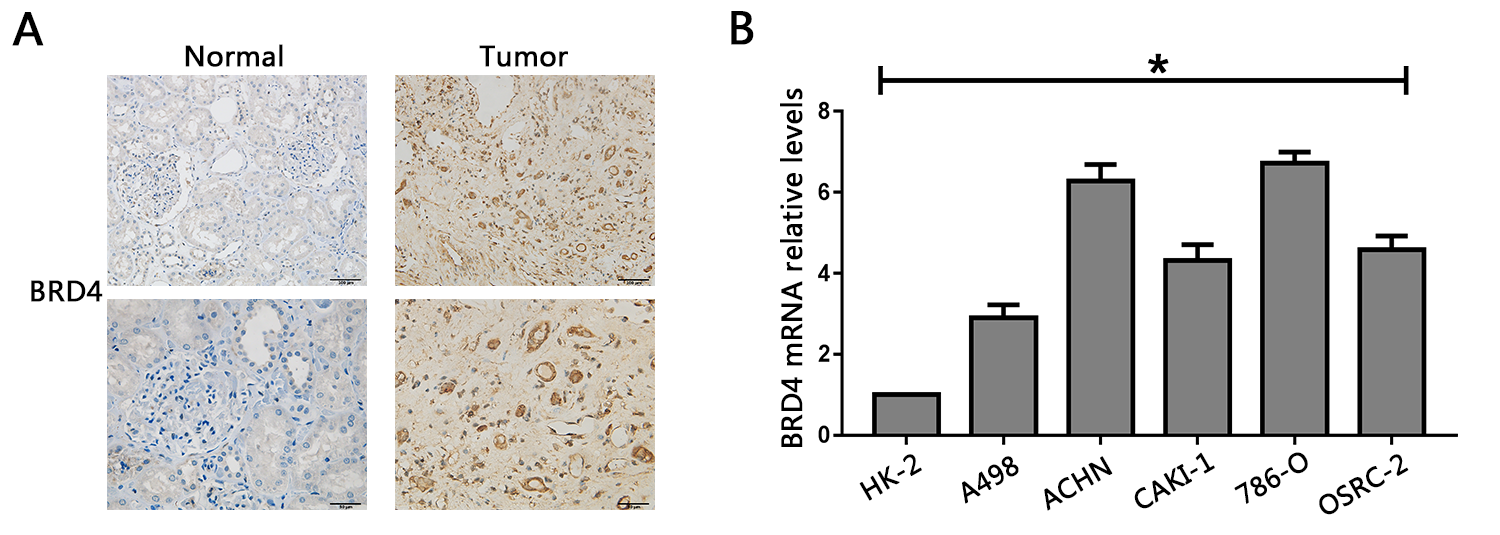

Supplement: Supplementary file 2 — Supplementary Figure 1 [file 41419_2020_2431_MOESM2_ESM.tif]

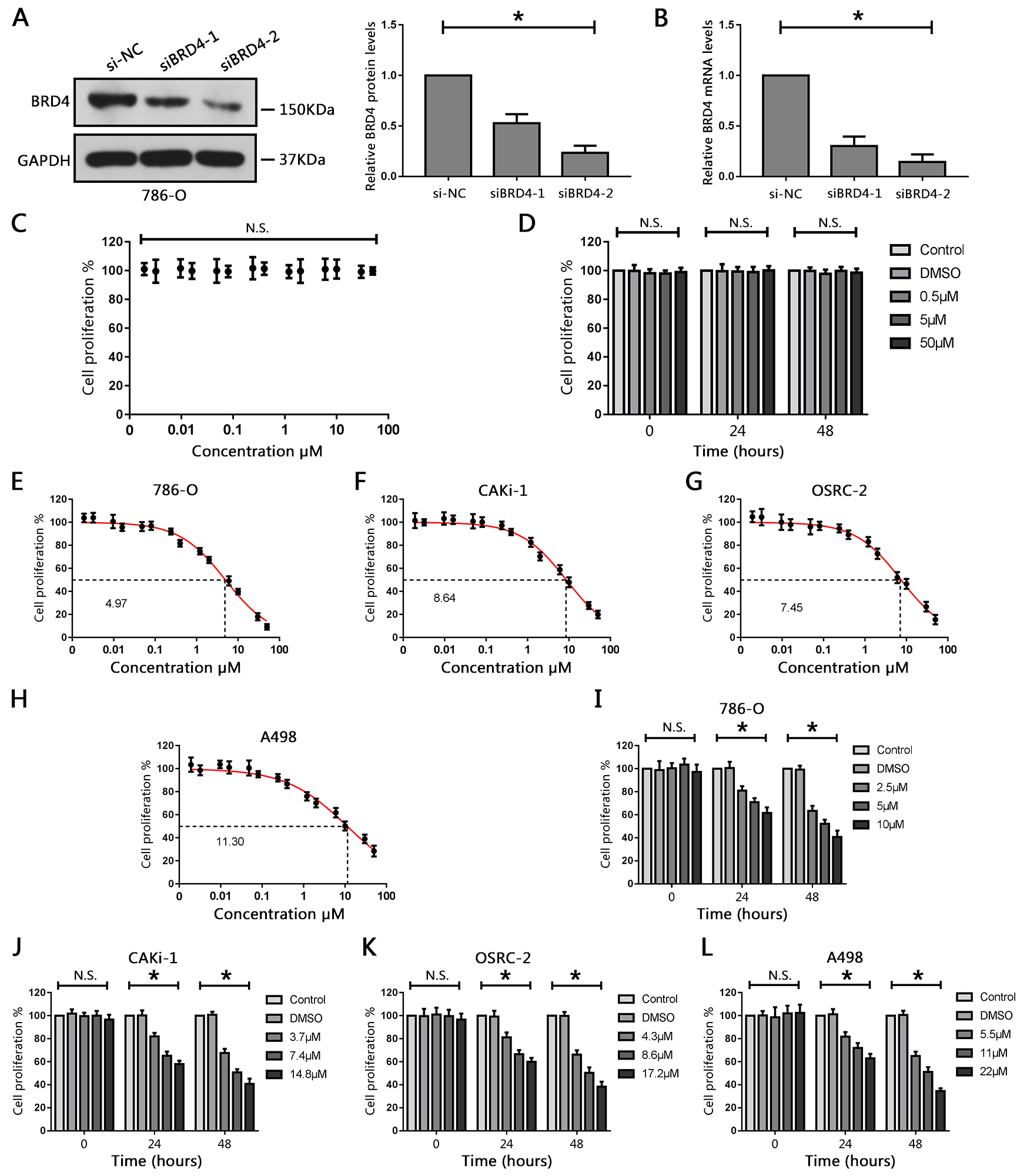

Supplement: Supplementary file 3 — Supplementary Figure 2 [file 41419_2020_2431_MOESM3_ESM.tif]

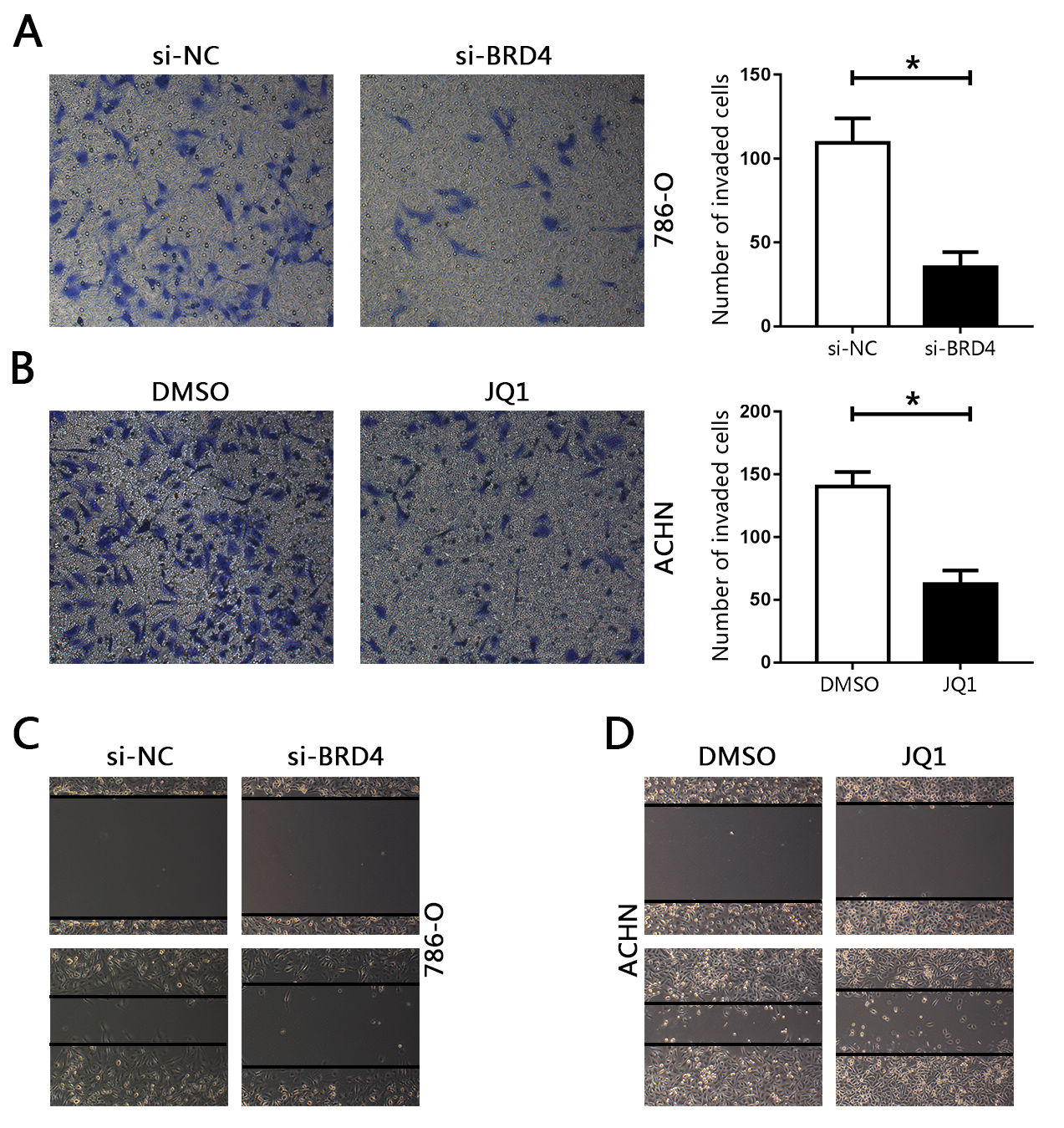

Supplement: Supplementary file 4 — Supplementary Figure 3 [file 41419_2020_2431_MOESM4_ESM.tif]

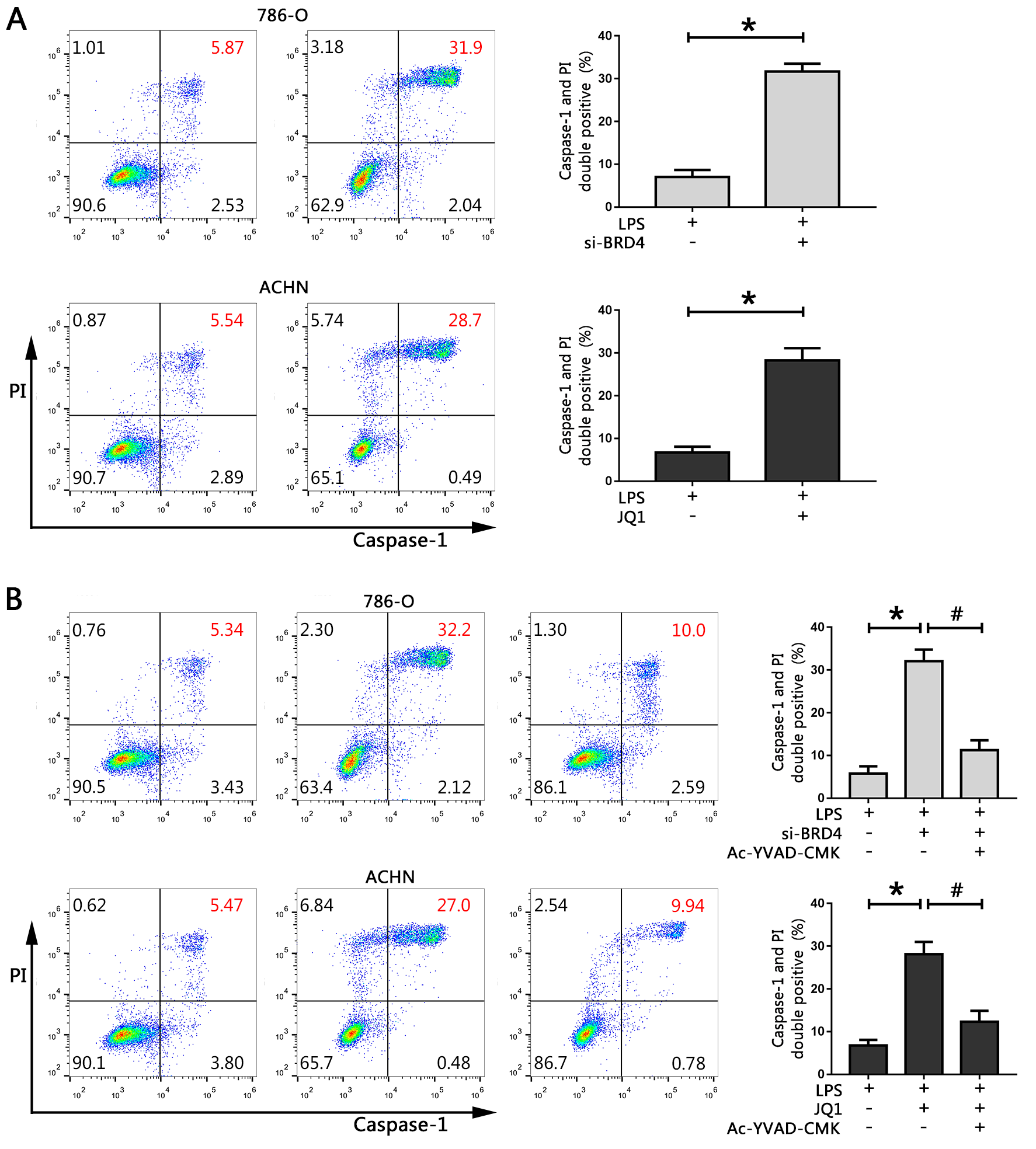

Supplement: Supplementary file 5 — Supplementary Figure 4 [file 41419_2020_2431_MOESM5_ESM.tif]

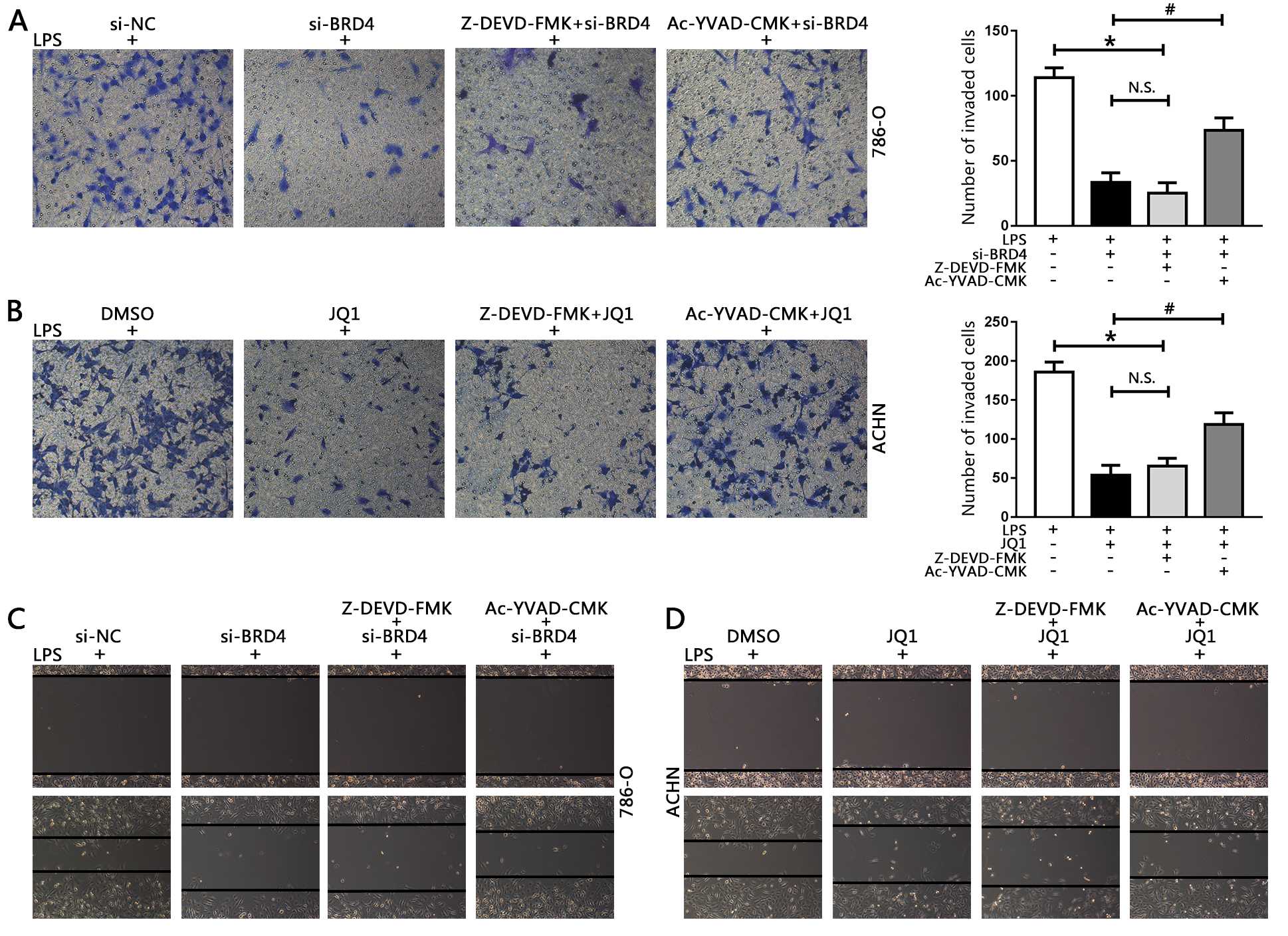

Supplement: Supplementary file 6 — Supplementary Figure 5 [file 41419_2020_2431_MOESM6_ESM.tif]

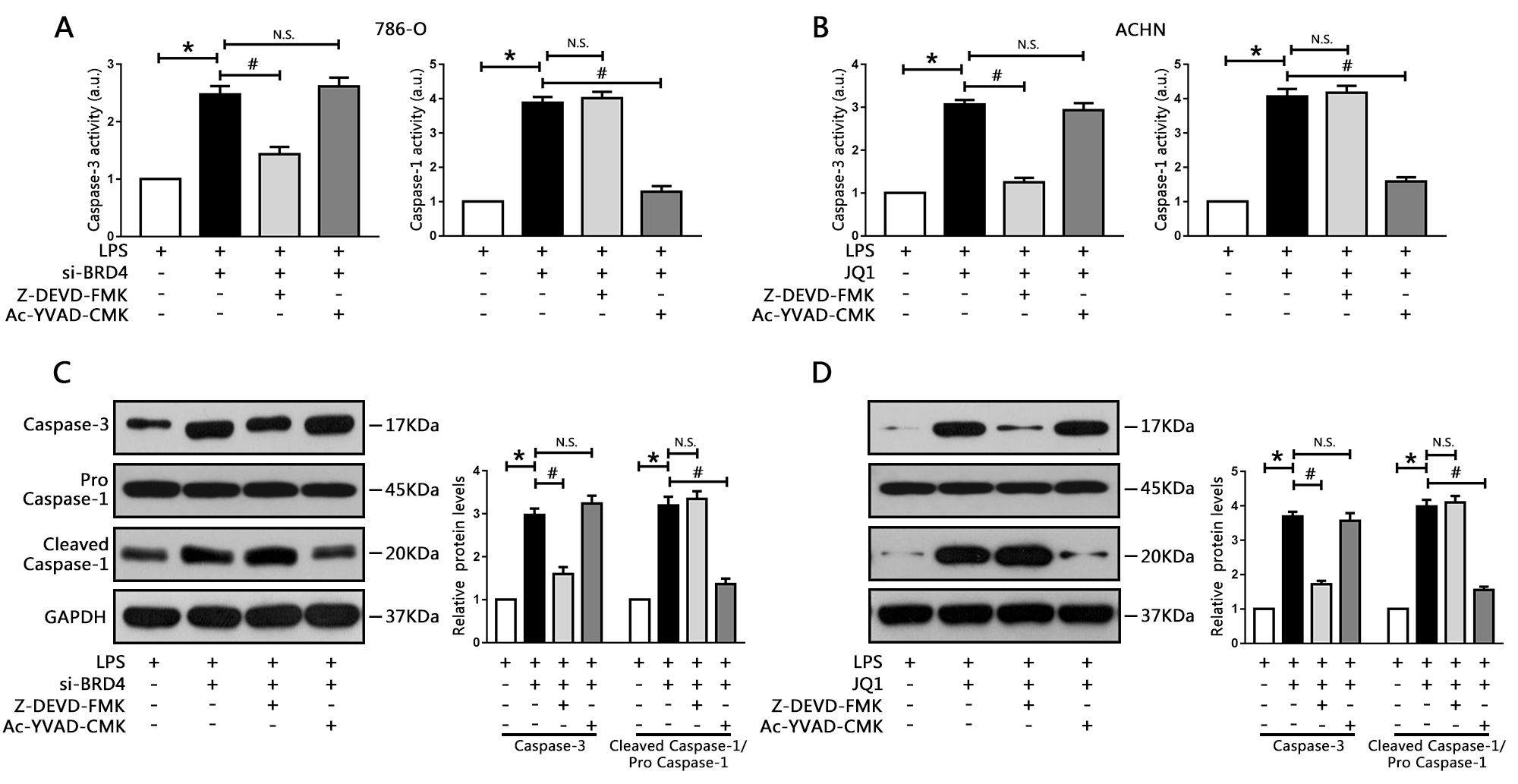

Supplement: Supplementary file 7 — Supplementary Figure 6 [file 41419_2020_2431_MOESM7_ESM.tif]

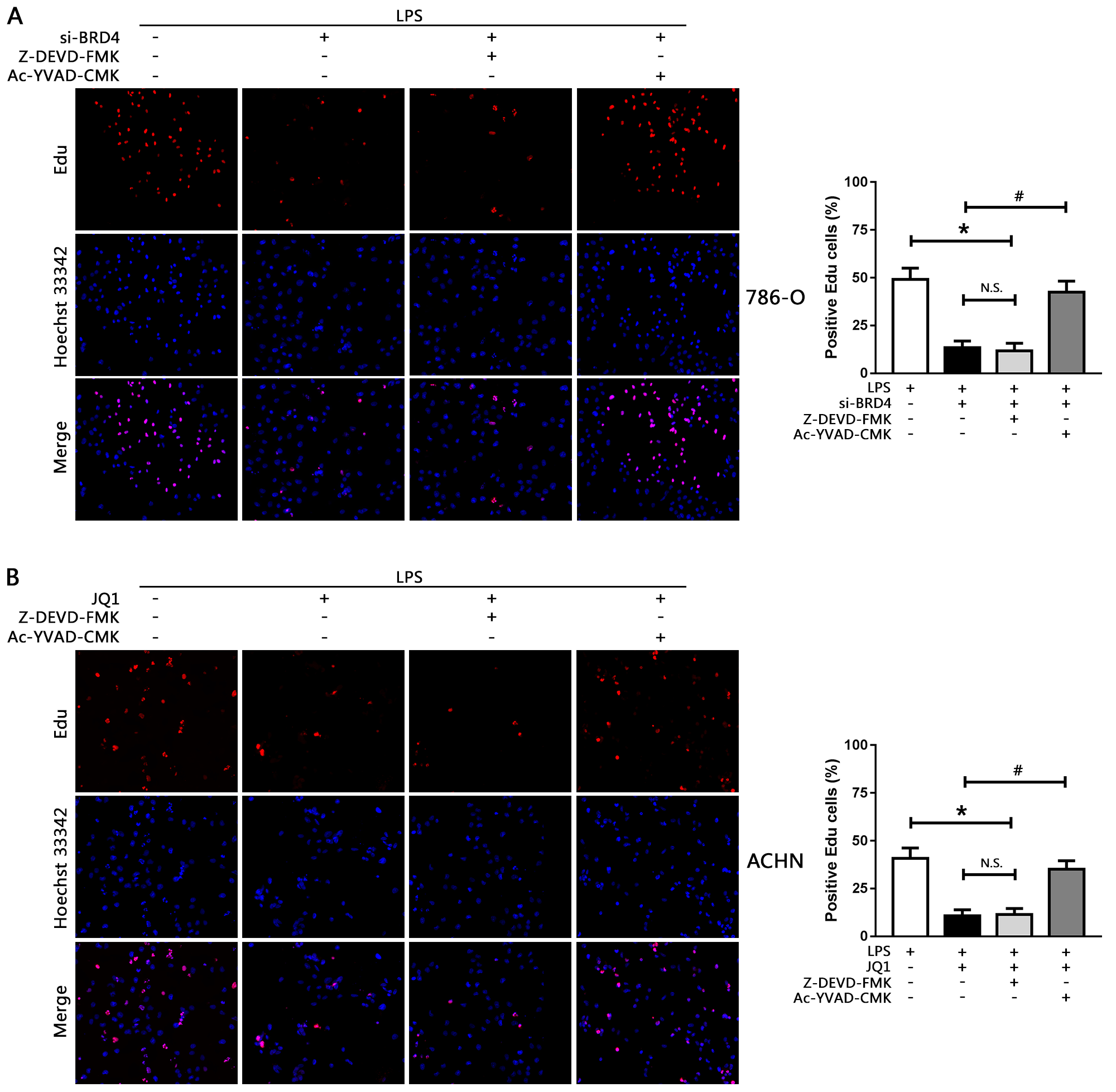

Supplement: Supplementary file 8 — Supplementary Figure 7 [file 41419_2020_2431_MOESM8_ESM.tif]

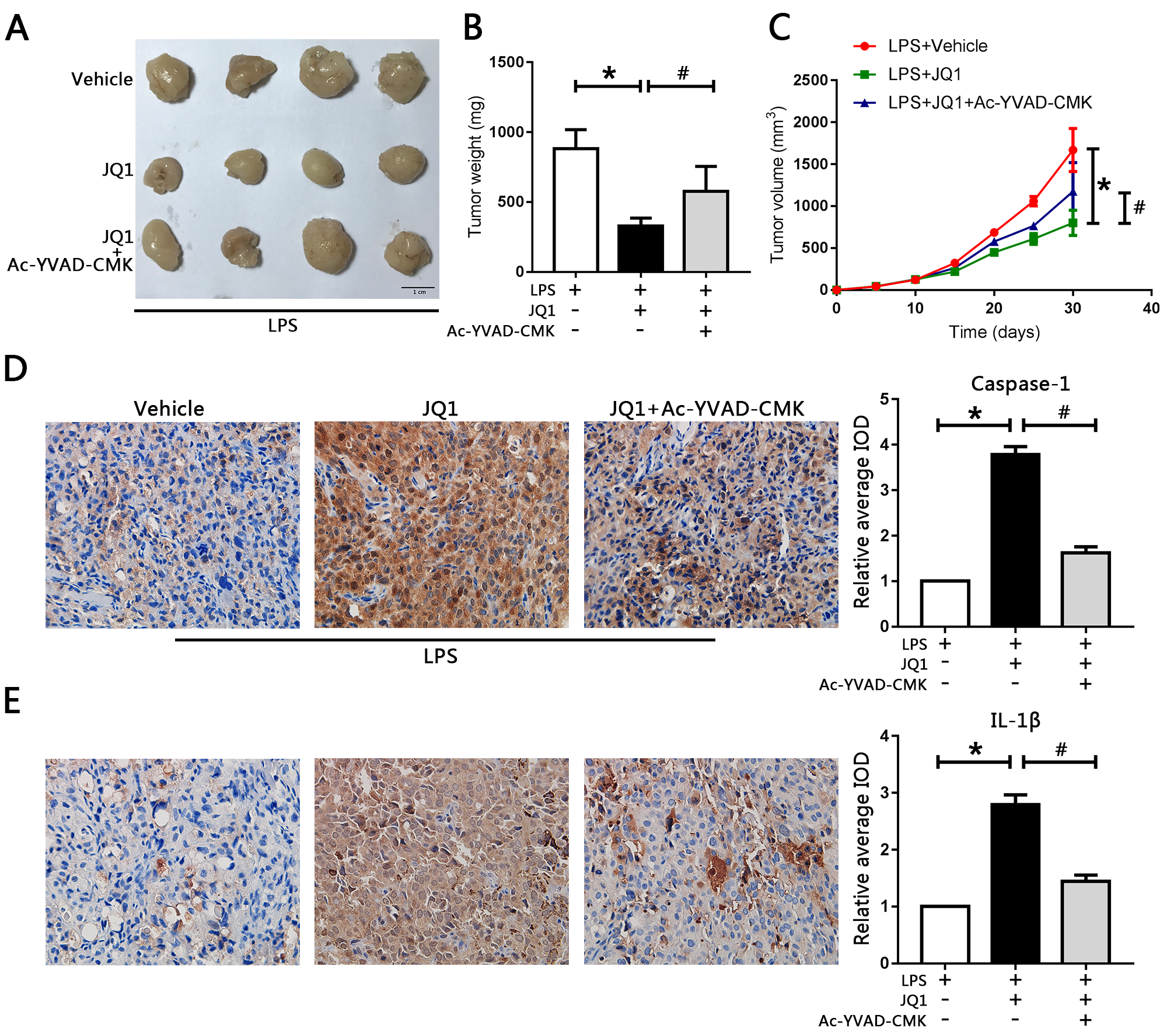

Supplement: Supplementary file 9 — Supplementary Figure 8 [file 41419_2020_2431_MOESM9_ESM.tif]

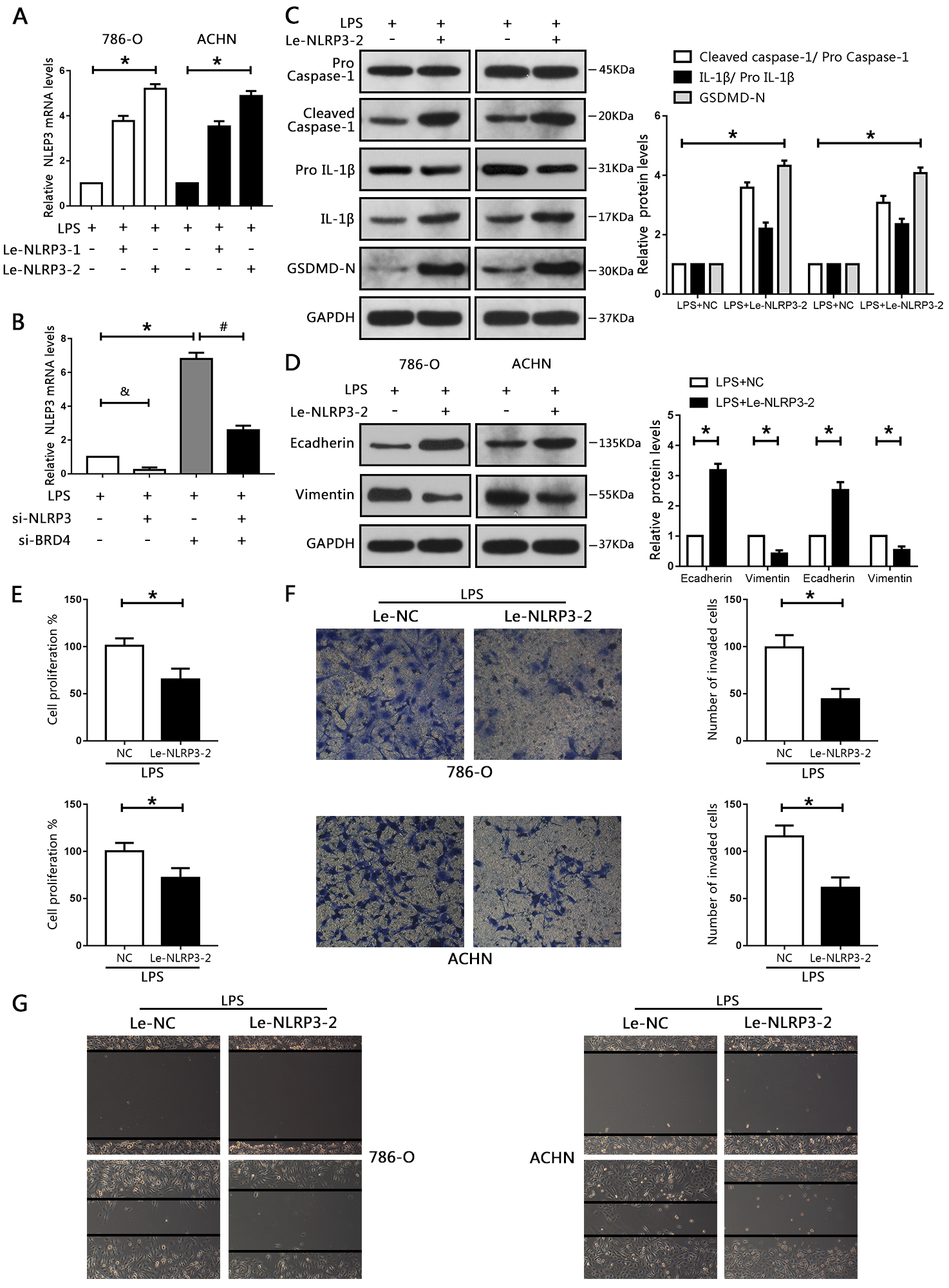

Supplement: Supplementary file 10 — Supplementary Figure 9 [file 41419_2020_2431_MOESM10_ESM.tif]

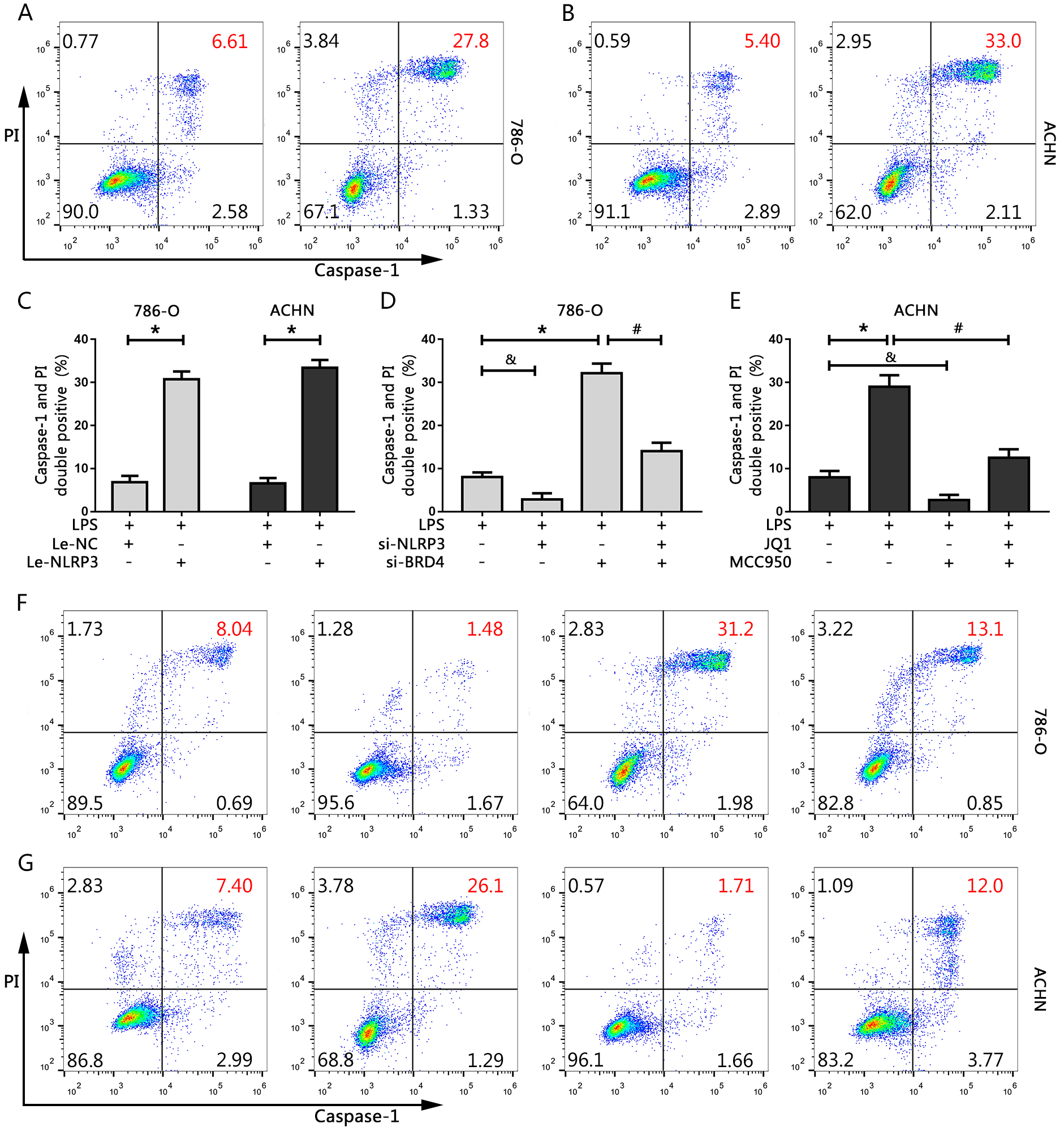

Supplement: Supplementary file 11 — Supplementary Figure 10 [file 41419_2020_2431_MOESM11_ESM.tif]

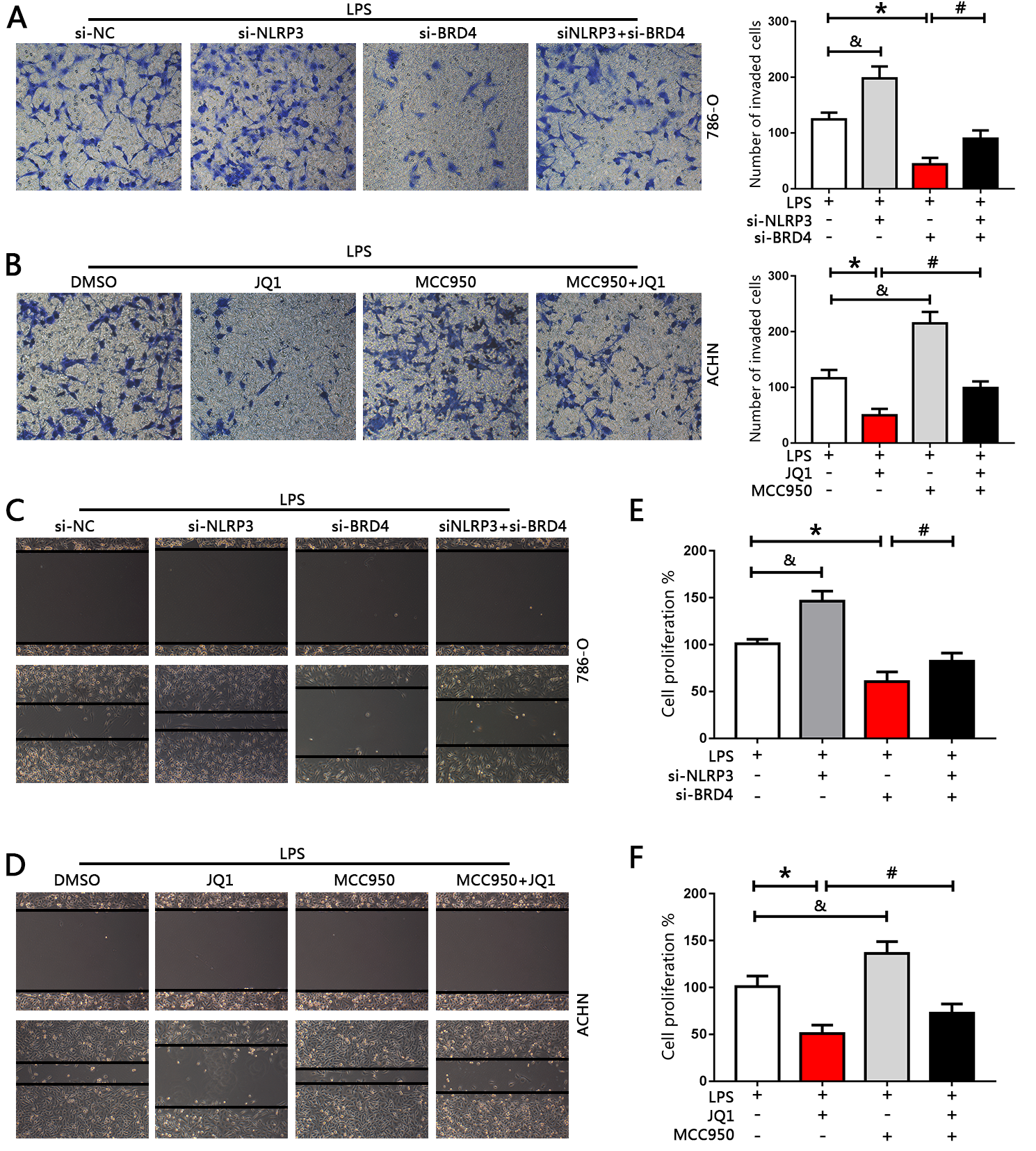

Supplement: Supplementary file 12 — Supplementary Figure 11 [file 41419_2020_2431_MOESM12_ESM.tif]

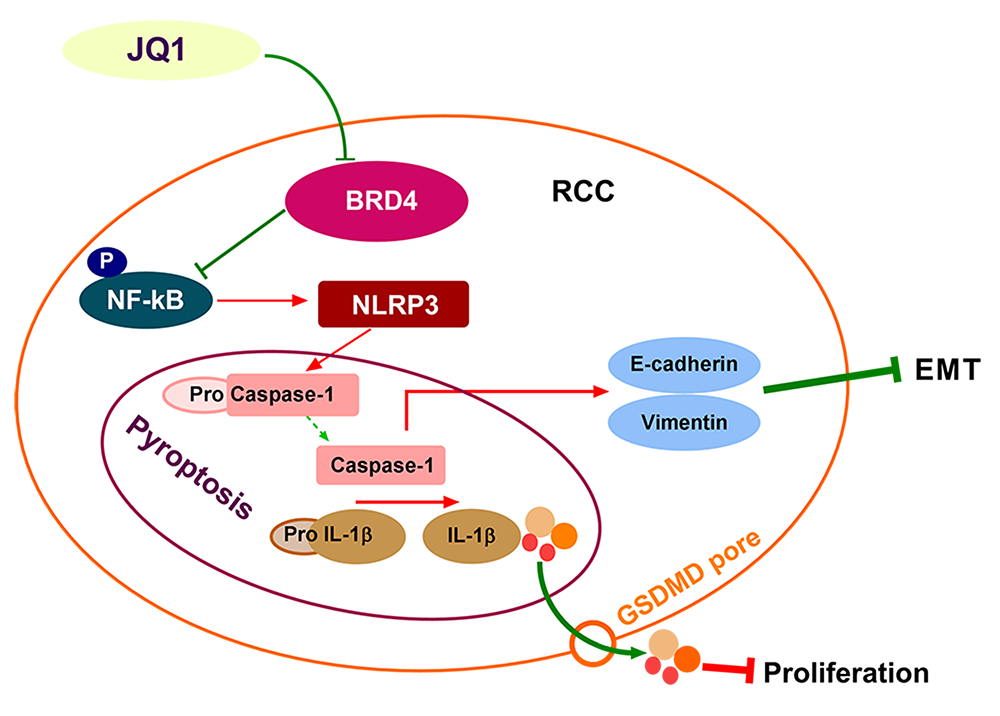

Supplement: Supplementary file 13 — Supplementary Figure 12 [file 41419_2020_2431_MOESM13_ESM.tif]
