# Supplementary material for: Inhibition of BRD4 prevents proliferation and epithelial–mesenchymal transition in renal cell carcinoma via NLRP3 inflammasome-induced pyroptosis
Source: Cell Death Dis. 2020 Apr 17;11(4):239. doi: 10.1038/s41419-020-2431-2 (PMC7165180; doi:10.1038/s41419-020-2431-2)
Supplement: Supplementary file 14 — Supplementary Table legends [file 41419_2020_2431_MOESM14_ESM.doc]

**Supplementary table legends**

Table 1: RT-PCR primer sequences were showed as followed.
