# Supplementary material for: Inhibition of BRD4 prevents proliferation and epithelial–mesenchymal transition in renal cell carcinoma via NLRP3 inflammasome-induced pyroptosis
Source: Cell Death Dis. 2020 Apr 17;11(4):239. doi: 10.1038/s41419-020-2431-2 (PMC7165180; doi:10.1038/s41419-020-2431-2)
Supplement: Supplementary file 15 — Supplementary Table 1 [file 41419_2020_2431_MOESM15_ESM.doc]

| Gene |  | Forward (5′-3′) | Reverse (5′-3′) |  |  | |  | |  |
| --- | --- | --- | --- | --- | --- | --- | --- | --- | --- |
| BRD4 | | TGAGTCGGAGGAAGAGGACAAGTG | CGCAGTGTGGACGGCTTCAG | | |  | |  | |
| NLRP3 | | CTTGCATCAGTATTGAGCACCA | CCAGTTTCTGCAGGTTACACT | | |  | |  | |
| GAPDH | | CAACGTGTCAGTGGTGGACCTG | GTGTCGCTGTTGAAGTCAGAGGAG | | |  | |  | |
